# A CRISPR array orchestrates virulence and host response in *Porphyromonas gingivalis*

**DOI:** 10.1128/spectrum.02834-25

**Published:** 2026-02-25

**Authors:** Muhammad Irfan, Ana Duran-Pinedo, Jose Solbiati, Fernanda Godoy Rocha, Frank C. Gibson, Jorge Frias-Lopez

**Affiliations:** 1 Department of Oral Biology, College of Dentistry, University of Florida, Gainesville, Florida, USA; University of the Pacific Arthur A Dugoni School of Dentistry, San Francisco, California, USA

**Keywords:** CRISPR, *Porphyromonas gingivalis*, virulence, regulation of gene expression

## Abstract

CRISPR–Cas systems are primarily recognized for their defensive role against foreign DNA. Recent studies, however, indicate involvement in regulatory functions. The persistence of a large spacer array without apparent phage targets in *Porphyromonas gingivalis* ATCC 33277 remains unexplained. This study demonstrates that deletion of the non-coding CRISPR array 30.1 in *P. gingivalis* ATCC 33277 results in increased biofilm formation, elevated virulence in a *Galleria mellonella* infection model, and significant alterations in the macrophage transcriptomic response. The ΔCRISPR-30.1 mutant forms twice as much biofilm as the wild type, induces 50% mortality in Galleria larvae within 130 h compared to 200 h for the wild type (*P* < 0.0001), and elicits a cytokine profile characterized by increased IL-6, CXCL1, CXCL2, and CXCL9 secretion. Dual RNA sequencing of THP-1 macrophages infected with wild-type and ΔCRISPR 30.1 strains reveals that the loss of the array activates bacterial metabolic and secretion pathways while suppressing host innate and adaptive immune signaling. Single-primer amplification (SPA) identifies numerous self-genome loci bound by individual 30.1 spacers, supporting a direct, spacer-mediated regulatory mechanism. These findings establish CRISPR array 30.1 as a previously unrecognized regulator of *P. gingivalis* physiology and host-pathogen interactions.

**IMPORTANCE:**

CRISPR–Cas systems are established as adaptive immune elements, yet spacer arrays without known targets are frequently observed in bacteria and often lack a defined function. In *P. gingivalis*, a keystone periodontal pathogen, a non-coding CRISPR array has been shown to regulate biofilm formation, virulence in an invertebrate model, and the macrophage transcriptional response. This expands the recognized functions of CRISPR arrays to include the direct regulation of bacterial physiology and the modulation of host immune responses, identifying CRISPR spacers as potential targets for antimicrobial interventions. Furthermore, elucidating the role of CRISPR arrays in *P. gingivalis* may have broader clinical implications, given the established associations between periodontal health and systemic inflammatory diseases. Targeting spacer arrays to modulate bacterial virulence could influence the management of these conditions and enhance the translational relevance of such therapeutic strategies.

## INTRODUCTION

Clustered Regularly Interspaced Short Palindromic Repeats and CRISPR-associated (CRISPR–Cas) systems typically consist of CRISPR arrays, non-coding DNA regions containing spacers, and their associated Cas proteins (1). These systems are primarily recognized for providing bacteria with adaptive immunity against bacteriophages and mobile genetic elements by capturing fragments of invading nucleic acids as spacers and using them to target future invasions (2–5). However, mounting evidence indicates the additional roles for CRISPR–Cas systems, extending beyond antiviral defense to influence bacterial physiology and virulence (3, 6–11). Non-canonical functions of CRISPR–Cas systems include biofilm formation, regulation of antibiotic resistance gene acquisition, DNA repair, modulation of competitive interactions among species, stress responses, and regulation of endogenous gene expression (5). In *Salmonella enterica* serovar Typhimurium, for example, the CRISPR–Cas system positively regulates key virulence determinants, such as antioxidant defenses and SPI-1 and SPI-2 pathogenicity islands, which are essential for bacterial invasion and intracellular survival (9). The precise molecular mechanisms underlying these regulatory roles remain largely unknown, although the self-targeting spacers, which match the sequences within the bacterial chromosome, are proposed to mediate such control (12–14). However, the extent to which CRISPR–Cas systems in bacteria, such as *Porphyromonas gingivalis*, serve regulatory roles remains poorly understood. *Porphyromonas gingivalis*, a keystone pathogen implicated in periodontal disease (15, 16), encodes multiple CRISPR–Cas systems, despite limited evidence of associated bacteriophages in the oral cavity (17–19). Although oral microbiome CRISPR loci are transcriptionally active, their spacers rarely match known oral viral sequences, suggesting roles beyond antiviral immunity (5, 18, 20). Specifically, *P. gingivalis* strain ATCC 33277 harbors four distinct CRISPR arrays (30.1, 36.1, 36.2, and 37) linked to type I-B, III, VI-B1, and VI-B2 Cas operons, respectively (3, 17). CRISPR array 30.1 alone contains 119 spacers, several of which demonstrate high similarity to endogenous bacterial sequences, indicating potential regulatory functions (3, 21, 22). A notable analysis demonstrated that CRISPR spacers in *P. gingivalis* often exhibit a high degree of sequence similarity to regions of its own chromosomal DNA, including IS elements. Specifically, Watanabe et al. reported that approximately 1.6% of all unique spacers characterized in their study were homologous to these IS regions, suggesting a significant role for CRISPR in limiting IS transposition and thus in regulating genetic diversity within the species (23). A review by Chopra et al. corroborates that the prevalence of CRISPR spacers targeting sequences within the genus *Porphyromonas* is markedly higher compared to targets from other genera, suggesting a specialized adaptation to its ecological niche (24). This contention is further supported by functional analyses that show a direct correlation between the presence of CRISPR spacers and pathogenicity-related genetic regions, suggesting that CRISPR spacers may safeguard essential genomic regions critical for virulence (3).

Transposon-sequencing analyses highlight the importance of CRISPR–Cas systems in epithelial colonization and abscess formation by *P. gingivalis* in murine models (21). Furthermore, metatranscriptomic studies have observed significant upregulation of CRISPR-associated genes during periodontal disease progression, underscoring potential roles in virulence modulation (25, 26). We recently demonstrated that the type I-B CRISPR–Cas system in *P. gingivalis* controls the expression of novel adhesins, directly affecting bacterial virulence both *in vitro* and *in vivo* (27, 28).

This study investigates the type I-B-associated CRISPR array 30.1 in *P. gingivalis* ATCC 33277. A deletion mutant (ΔCRISPR 30.1) was constructed to assess the role of the array in bacterial physiology and virulence. The removal of the CRISPR array 30.1 increased biofilm formation, enhanced virulence in a *Galleria mellonella* infection model, and induced significant changes in the host macrophage transcriptome. Macrophages play a myriad of functions in the host, including supporting tissue homeostasis during health, while in response to infection, these cells provide innate immune sensing, phagocytosis, and expression of an array of inflammatory mediators to aid in controlling disease. In the context of periodontal disease, macrophages comprise 5–30% of the inflammatory infiltrate observed (29). Furthermore, depletion of these cells led to an inability of the host to develop oral bone loss following *P. gingivalis* exposure (30). Thus, we chose to focus our investigations using macrophages as a model.

Single-primer amplification (SPA) mapped spacers to specific self-genomic loci within the *P. gingivalis* chromosome. These loci are enriched for genes involved in metabolism, DNA repair, stress responses, and regulatory functions, supporting a model in which self-targeting spacers modulate bacterial gene expression to regulate virulence and survival.

These results demonstrate that CRISPR arrays act as master regulators, coordinating bacterial virulence strategies and host-pathogen interactions. This finding broadens the conventional understanding of CRISPR–Cas systems, emphasizing their essential role in bacterial physiology and pathogenicity. The data also suggest new therapeutic opportunities to target pathogenic bacteria by disrupting CRISPR-mediated gene regulation.

## RESULTS

### Deletion of CRISPR array 30.1 enhances biofilm formation and increases virulence in a *Galleria mellonella* model

**Fig 1:**
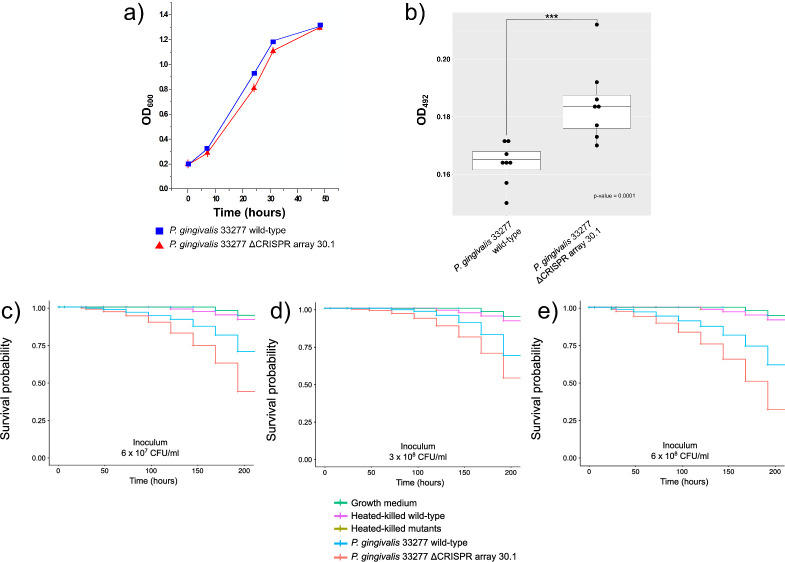
Impact of CRISPR array 30.1 deletion on growth, biofilm formation, and virulence of *P. gingivalis* ATCC 33277. (**a**) Growth curve comparing planktonic growth of wild-type and ΔCRISPR array 30.1 mutant strains. (**b**) Biofilm biomass quantification of wild-type and ΔCRISPR array 30.1 mutant strains grown on microtiter plates, assessed by safranin staining (OD at 492 nm). (**c–e**) Kaplan–Meier survival analysis of *Galleria mellonella* larvae injected with different concentrations of wild-type or ΔCRISPR array 30.1 mutant strains: (**c**) 6 × 10^7^ CFU/mL; (**d**) 3 × 10^8^ CFU/mL; and (**e**) 6 × 10^8^ CFU/mL. Survival differences between groups were statistically significant (*P* < 0.0001). Controls represent larvae injected with PBS only.

Deletion of CRISPR array 30.1 in *P. gingivalis* ATCC 33277 did not significantly affect planktonic cell growth compared to the wild type (Fig. 1a). Both growth curves showed a similar shape for a period of 50 h, reaching the stationary phase at an OD_600_ of 1.3. However, assessment of biofilm formation on microtiter plates showed that the ΔCRISPR array 30.1 mutant exhibited significantly increased biofilm biomass, as quantified by safranin staining and optical density measurement at 492 nm (mean OD₄₉₂ values of 0.165 in the wild type vs 0.185 in the mutant) (Fig. 1b).

To directly evaluate the contribution of CRISPR array 30.1 to bacterial virulence, groups of 15 *Galleria mellonella* larvae were challenged by injection with varying bacterial concentrations (ranging from 10^7^ to 10^8^ CFU/mL). Mortality rates were significantly higher (*P* < 0.0001) in larvae infected with the ΔCRISPR array 30.1 mutant. All control conditions (growth medium, heated-killed wild-type, and heated-killed mutants) showed 100% survival throughout the 200-hour period. At a bacterial load of 6 × 10^8^ CFU/mL, the mutant induced 50% larval mortality by 130 h, compared to 38% mortality by 200 h for the wild-type strain (Fig. 1e). Similarly, at lower inocula (6 × 10^7^ and 3 × 10^8^ CFU/mL), larvae infected with the mutant exhibited higher survival probabilities compared to those infected with the wild-type strain over 200 h (Fig. 1). Control larvae showed no mortality over the 7-day observation period (survival probability of 1) (Fig. 1).

### Impact of CRISPR array 30.1 deletion on cytokine and chemokine production by THP-1 cells

The impact of CRISPR region deletion on the intracellular metabolism of *P. gingivalis* and the host immune response was evaluated. Macrophage-like THP-1 cells, a human leukemia monocytic cell line treated with PMA, were used to measure cytokine and chemokine levels in cell culture supernatants at 2 and 6 h post-infection using Luminex multiplex assays. As indicated above, macrophages are key inflammatory cells found in periodontal disease lesions and participate in oral bone loss (29, 30). Our THP-1 cells mounted robust, reproducible responses that cleanly separated experimental conditions, with strong shift into a classically inflammatory state. This mirrors *in vivo* observations that *P. gingivalis* recruits and activates inflammatory macrophages and that their effector outputs (NO, proinflammatory cytokines, and chemokines) track with infection burden and bone resorption (30), thereby validating macrophages as an informative host context for these response experiments. Upon stimulation, PMA-differentiated THP-1 macrophages rapidly engaged NFκB/MAPK signaling and produced a characteristic M1 cytokine/chemokine program (TNFα, IL1β, IL6, IL12p70, CCL2), consistent with early pathway engagement and induction of canonical M1 mediators.

**Fig 2:**
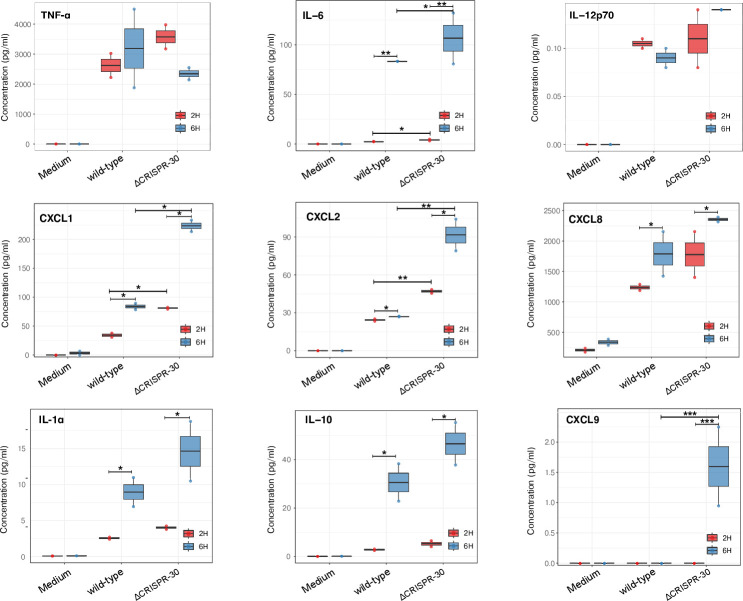
Deletion of the CRISPR array 30.1 alters cytokine and chemokine secretion by THP-1 macrophages upon *P. gingivalis* infection. THP-1 cells were infected with wild-type or ΔCRISPR 30.1 P*. gingivalis* (multiplicity of infection = 100), and supernatants were collected at 2 h and 6 h post-infection. Cytokine and chemokine levels were measured by Luminex multiplex assay. Data are mean ± SD of three independent experiments (*n* = 3). Statistical differences were evaluated using a false discovery rate (FDR) value of <0.05 for multiple-comparison corrections comparisons test: * 0.01 < *P* ≤ .05, ** 0.001 < *P* ≤ .01, *** *P* ≤ 0.001 (see Jupyter notebook for details).

Most cytokines exhibit higher levels at 6 h compared to 2 h, with statistical significance primarily observed at the 6-hour time point for most markers. Infection with the ΔCRISPR array 30.1 mutant resulted in significantly increased secretion of IL-6 and chemokines MIP-2α (CXCL2), CXCL1, and CXCL9 (Fig. 2; Fig. S1). IL-6 showed a roughly 32% increase between the wild-type (~83 pg/mL) and the ΔCRISPR mutant (~110 pg/mL) at 6 h. CXCL9 exhibits the strongest statistical significance in both time comparisons, with a substantial fold change at 6 h (wild type: ~0.05 pg/mL vs ΔCRISPR mutant: ~1.8 pg/mL). The predominance of significance at 6 h versus 2 h indicates transcriptional amplification in THP-1 macrophages, consistent with robust NFκB/MAPK activation and engagement of a CXCR3 ligand chemokine program. By 6 h, upstream inflammatory signals (classically NFκB and MAPK) lead to production of IL6 and CXCL1/2 into the cell culture supernatant fluids. The rise in CXCL9 indicates explicit activation of the CXCR3 ligand chemokine axis (CXCL9/10/11 genes controlled by STAT1/IRF/NFκB), which recruits CXCR3+ effector cells, such as Th1 T lymphocytes and macrophages (31).

CXCL1 and CXCL2 primarily recruit neutrophils, while CXCL9 predominantly attracts T cells, natural killer (NK) cells, and dendritic cells to sites of inflammation. IL-10, IL-1α, and IL-8 (CXCL8) levels also increased, but did not reach statistical significance. CXCL1 showed the most dramatic increase (wild type ~35 pg/mL vs ΔCRISPR mutant ~240 pg/mL) at 6 h (Fig. S1).

TNF-α showed very high concentrations (~2,500–4,000 pg/mL for both strains), but there was no apparent significant difference between the wild type and ΔCRISPR mutant.

### Impact of CRISPR array 30.1 deletion on host-pathogen transcriptomic profiles

**Fig 3:**
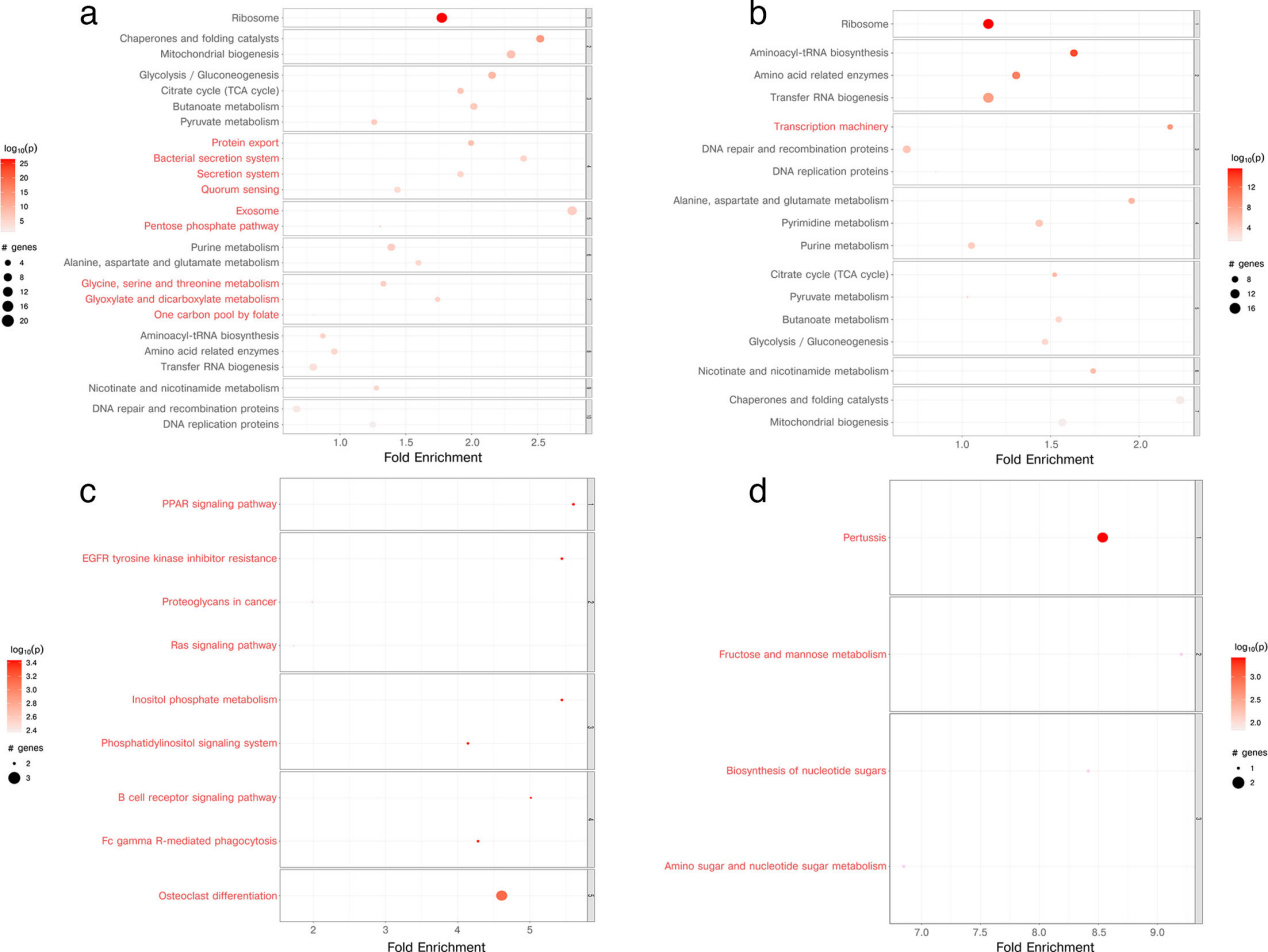
PathfindR-derived KEGG pathway enrichment in *P. gingivalis* and THP-1 cells following infection with the ΔCRISPR 30.1 mutant. Bubble plots display enriched pathways clustered by functional category. The x-axis shows fold enrichment, the y-axis lists KEGG pathway names, bubble size reflects the number of differentially expressed genes (DEGs) in each pathway, and bubble color indicates statistical significance (–log₁₀ of the lowest *P*-value; deeper red denotes more substantial enrichment). Red pathway labels denote clusters in which all constituent genes are upregulated in the ΔCRISPR 30.1 mutant compared to wild type. (**a**) KEGG pathway enrichment in *P. gingivalis* at 2 h post-infection. (**b**) KEGG pathway enrichment in *P. gingivalis* at 6 h post-infection. (**c**) KEGG pathway enrichment in THP-1 cells at 2 h post-infection. (**d**) KEGG pathway enrichment in THP-1 cells at 6 h post-infection.

Dual transcriptomic analysis, capturing gene expression changes in THP-1 cells and intracellular *P. gingivalis*, revealed extensive and quantitatively significant pathway alterations that demonstrate the profound metabolic reprogramming occurring in both bacterial and host cells. The magnitude of gene involvement varied considerably across pathways, as indicated by the dot sizes representing the number of genes, with some pathways encompassing substantial gene sets (Fig. 3).

Within each time point, expression profiles of wild type and ΔCRISPR array 30.1 mutant strains were highly similar (Fig. S2a). In contrast, host cell transcriptional profiles were strongly influenced by incubation duration, with greater differences observed between 2- and 6-hour time points than those induced by bacterial strain type (Fig. S2b).

At 2 h post-infection, ribosomal pathways showed the highest fold enrichment in bacterial cells (~2.2–2.5×), representing the most significantly activated pathway, with additional notable activation of chaperones and folding catalysts as well as the mitochondrial biogenesis pathway (Fig. 3a). By 6 h, ribosomal function remained highly enriched (~2.0× fold), accompanied by strong activation of aminoacyl-tRNA biosynthesis (~1.8× fold enrichment), transfer RNA biogenesis, and transcription machinery, indicating sustained protein synthesis machinery upregulation in the ΔCRISPR mutant (Fig. 3b). The ΔCRISPR mutant showed enhanced activation of key metabolic and biosynthetic pathways, including core metabolism (TCA cycle, glycolysis/gluconeogenesis, pyruvate, and butanoate metabolism) and nucleotide and amino acid synthesis pathways at both 2 and 6 h (Fig. 3a and b; Fig. S3b). Notably, at 2 h post-infection, unique activation of secretion-associated pathways (protein export, bacterial secretion systems, quorum sensing, and outer membrane vesicle formation) was observed (Fig. 3a; Fig. S3b).

Host cell responses exhibited even more dramatic quantitative changes, with the PPAR signaling pathway showing the highest fold enrichment at 2 h (~5-fold), followed by EGFR tyrosine kinase inhibitor resistance pathways (~4-fold enrichment). Additional pathways include proteoglycans in cancer, inositol phosphate metabolism, and phosphatidylinositol signaling systems, all showing significant statistical activation based on log₁₀(*P*) values (Fig. 3c). Most strikingly, the 6-hour host response revealed pertussis pathways with extremely high fold enrichment (~8.5×), representing the most dramatic pathway activation observed across all conditions and time points (Fig. 3d). Carbohydrate metabolism pathways, including fructose and mannose metabolism and biosynthesis of nucleotide sugars, appeared as distinct and separate regulatory modules, with the overall scale reaching fold enrichments up to 9-fold.

The network analysis revealed additional layers of complexity that were not captured by pathway enrichment alone. The host pathway network demonstrated extensive interconnections between immune signaling, metabolic regulation, and cellular processes, with temporal specificity clearly delineated through color coding: purple dots representing 2-hour-specific pathways, blue dots for 6-hour-specific responses, and yellow dots for common pathways, overlaid with upregulated (green) and downregulated (red) gene expression patterns. Central hub pathways emerged as major nodes with multiple connections, particularly within immune signaling clusters, suggesting coordinated regulatory control across biological processes (Fig. S3a). The host THP-1 cells showed activation of NF-κB signaling and platelet activation at both time points (Fig. S3a). At 2 h, host pathways involved in immune signaling (B cell receptor signaling, Fc gamma receptor-mediated phagocytosis), phospholipid signaling, intracellular calcium mobilization, growth factor signaling (EGFR inhibitor resistance, Ras signaling), fatty acid regulation (PPAR signaling), and osteoclast differentiation were highly activated (Fig. 3c; Fig. S3a). At 6 h, carbohydrate metabolism and immune modulation pathways (amino sugar metabolism, nucleotide sugar synthesis, and pertussis host-pathogen interactions) predominated (Fig. 3d; Fig. S3a).

The bacterial gene network presented an even more comprehensive picture, with hundreds of individual genes mapped to specific pathways, revealing that this represents massive metabolic reprogramming rather than modest pathway adjustments (Fig. S3b). Gene regulation patterns showed coordinated upregulation (green) and downregulation (red) within pathway clusters, with related metabolic pathways clustering together (e.g., ribosome clusters, amino acid metabolism clusters). The density and complexity of the bacterial network exceeded that of the host response, indicating that CRISPR array deletion triggers extensive bacterial transcriptional restructuring. Disease-related pathways, including those related to COVID-19 and cancer, appeared in the host network, but were not addressed in the original text, suggesting broader implications for pathogen-host interactions in disease contexts. This network-level analysis demonstrates that the ΔCRISPR mutant orchestrates coordinated regulation across multiple biological processes, representing a fundamental shift in bacterial physiology and host-pathogen dynamics rather than isolated pathway alterations.

### Identification of CRISPR array 30.1 spacer genomic targets via single-primer amplification (SPA)

**Fig 4:**
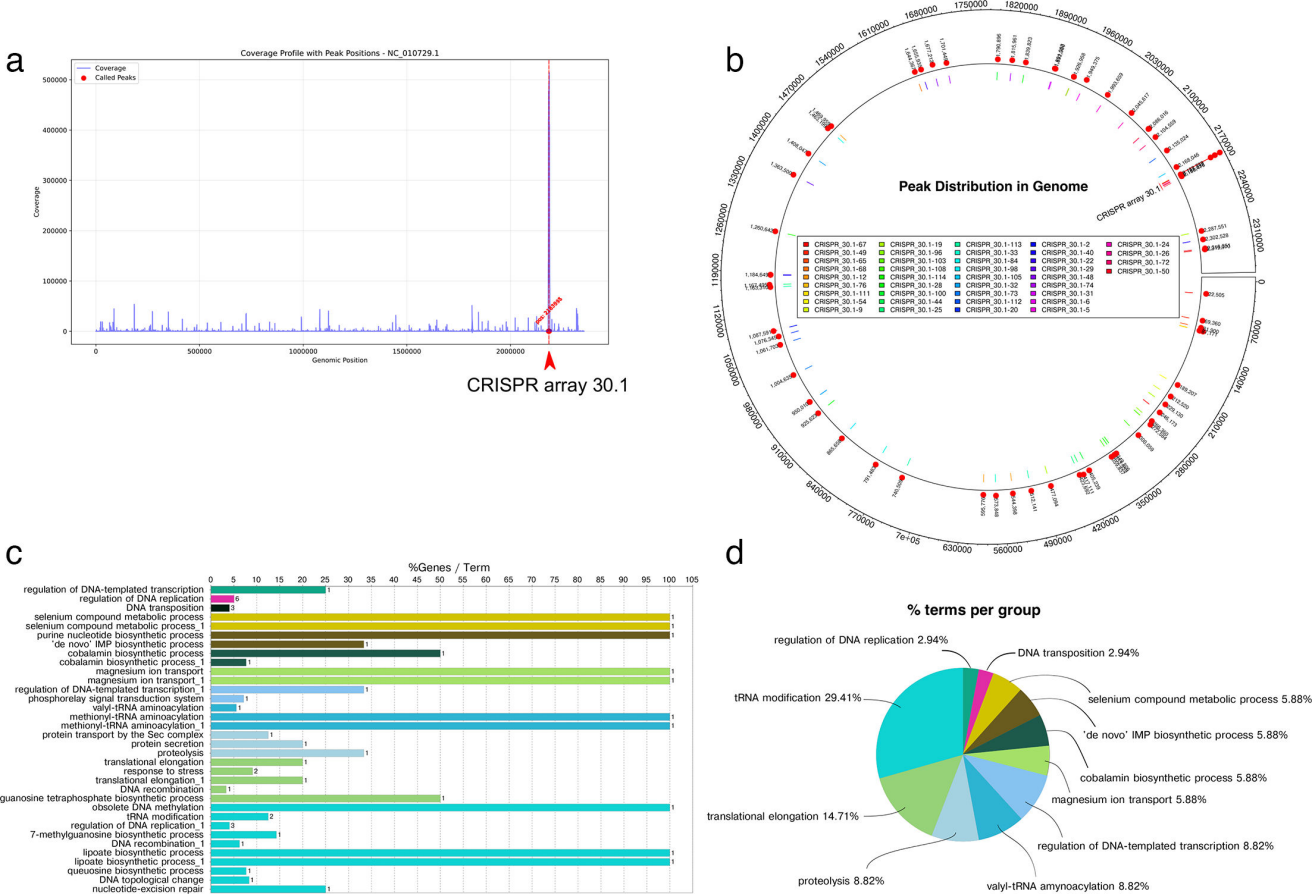
Single-primer amplification (SPA) mapping and PathfindR enrichment. (**a**) SPA positive control: PCR amplification using a mixture of all CRISPR array 30.1 spacers as primers against wild-type genomic DNA yields a single, dominant amplicon corresponding to the CRISPR array itself, confirming the specificity of the primers. (**b**) Genome-wide SPA peak distribution: Mapped SPA amplicon counts across the *P. gingivalis* chromosome reveal discrete binding sites (peaks) in both coding and intergenic regions, indicating self-targeting by array 30.1 spacers. (**c**) KEGG enrichment, % genes per term: PathfindR analysis of SPA-identified gene targets displays enriched KEGG pathways, plotted as the percentage of total differentially targeted genes annotated to each pathway. Bubble size corresponds to –log₁₀(*P*-value). (**d**) KEGG enrichment, % terms per cluster: The same enrichment results, grouped by functional clusters, are plotted as the percentage of enriched pathways within each cluster. Bubble size indicates the number of pathways in that cluster, and color intensity reflects statistical significance (–log₁₀(*P*-value)).

To further elucidate the regulatory mechanisms by which CRISPR spacers modulate bacterial metabolism, we performed SPA using 119 individual spacers from CRISPR array 30.1 as primers against genomic DNA from the ΔCRISPR array 30.1 mutant. We sequenced and mapped amplified products to reveal specific genomic targets. When we used positive controls with wild-type genomic DNA, we predominantly amplified the CRISPR array 30.1 region itself. As expected, the coverage profile demonstrates the expected amplification pattern when using wild-type DNA as a control (Fig. 4a).

SPA produced two distinct amplification patterns: genuine specific binding (Fig. S4a and b) and non-specific binding, characterized by diverse PCR products (Fig. S4a and c).

Overall, 41 out of 119 spacers (109 primers) yielded specific PCR products, while 68 spacers produced non-specific binding patterns. SPA targets were distributed genome-wide, encompassing both gene-coding and intergenic regions (Fig. 4b; Table 1). SPA results showed that targets are distributed across the entire circular chromosome, rather than being clustered. Targeted genes included those involved in membrane transport (sulfate permease, magnesium transporter, transporter, transporter substrate-binding domain-containing protein, and ABC-F family ATP-binding cassette domain-containing protein), DNA replication, repair, and modification (excinuclease ABC subunit UvrA, site-specific DNA-methyltransferase, RecQ family ATP-dependent DNA helicase, and UvrD-helicase domain-containing protein), cofactor and vitamin biosynthesis (cob(I)yrinic acid a,c-diamide adenosyltransferase [cobalamin biosynthesis], lipoyl synthase, selenide, and water dikinase SelD [selenium metabolism]), conjugation and mobile genetic elements (conjugative transposon protein TraK, conjugal transfer protein MobC, and IS5 family transposase), phosphatases and hydrolases (phosphatase PAP2 family protein, alkaline phosphatase family protein, MBL fold metallo-hydrolase), central metabolism and energy (fumarate reductase/succinate dehydrogenase flavoprotein subunit, aldehyde dehydrogenase family protein, NAD(P)-dependent oxidoreductase), protein synthesis and processing (methionine—tRNA ligase, elongation factor G, RsiV family protein, S46 family peptidase, prolyl oligopeptidase family serine peptidase, signal peptidase I), transcriptional regulation (helix-turn-helix transcriptional regulator, response regulator transcription factor), and CRISPR–Cas system (type VI-B CRISPR-associated RNA-guided ribonuclease Cas13b) (Table 1).

**TABLE 1:** Spacer targets identified by SPA

Self-target genes	
Locus tag	Gene function
PGN_RS00090	sulfate permease
PGN_RS00290	conjugative transposon protein TraK
PGN_RS00360	conjugal transfer protein MobC
PGN_RS00390	helix-turn-helix transcriptional regulator
PGN_RS00830	DUF3868 domain-containing protein
PGN_RS01085	hypothetical protein
PGN_RS01190	magnesium transporter
PGN_RS01215	lytic transglycosylase domain-containing protein
PGN_RS01350	methionine—tRNA ligase
PGN_RS01555	IS5 family transposase
PGN_RS01580	transglutaminase domain-containing protein
PGN_RS01820	transporter
PGN_RS02235	TGS domain-containing protein
PGN_RS02370	fumarate reductase/succinate dehydrogenase flavoprotein subunit
PGN_RS02500	phosphatase PAP2 family protein
PGN_RS02595	alkaline phosphatase family protein
PGN_RS03450	aldehyde dehydrogenase family protein
PGN_RS03980	IS5 family transposase
PGN_RS04115	site-specific DNA-methyltransferase
PGN_RS04305	response regulator transcription factor
PGN_RS04635	DUF4252 domain-containing protein
PGN_RS04680	phosphoribosylaminoimidazolesuccinocarboxamide synthase
PGN_RS06040	cob(I)yrinic acid a%2Cc-diamide adenosyltransferase
PGN_RS06290	excinuclease ABC subunit UvrA
PGN_RS06315	T9SS type A sorting domain-containing protein
PGN_RS07005	lipoyl synthase
PGN_RS07045	S46 family peptidase
PGN_RS07160	RsiV family protein
PGN_RS07255	carboxypeptidase-like regulatory domain-containing protein
PGN_RS07620	transporter substrate-binding domain-containing protein
PGN_RS07720	type VI-B CRISPR-associated RNA-guided ribonuclease Cas13b
PGN_RS07825	IS5 family transposase
PGN_RS08050	selenide%2C water dikinase SelD
PGN_RS08055	prolyl oligopeptidase family serine peptidase
PGN_RS08250	RecQ family ATP-dependent DNA helicase
PGN_RS08615	RHS repeat-associated core domain-containing protein
PGN_RS08870	elongation factor G
PGN_RS08945	NAD(*P*)-dependent oxidoreductase
PGN_RS09215	signal peptidase I
PGN_RS09725	UvrD-helicase domain-containing protein
PGN_RS09800	ABC-F family ATP-binding cassette domain-containing protein
PGN_RS09805	tetratricopeptide repeat protein
PGN_RS12105	hypothetical protein
PGN_RS12170	MBL fold metallo-hydrolase

Self-target intergenic regions			
Flanking gene left	Gene function flanking gene left	Flanking gene right	Gene function flanking gene right
PGN_RS00390	helix-turn-helix transcriptional regulator	PGN_RS00395	DUF3945 domain-containing protein
PGN_RS00920	LysM peptidoglycan-binding domain-containing protein	PGN_RS11000	DUF1661 domain-containing protein
PGN_RS01005	hypothetical protein	None	None
PGN_RS01765	4'-phosphopantetheinyl transferase superfamily protein	PGN_RS01770	thioredoxin domain-containing protein
PGN_RS01860	PDZ domain-containing protein	PGN_RS01865	CinA family protein
PGN_RS02075	hypothetical protein	None	None
PGN_RS03240	signal peptide peptidase SppA	PGN_RS11465	DUF1661 domain-containing protein
PGN_RS03770	IS982-like element IS195 family transposase	PGN_RS03775	trigger factor
PGN_RS04540	hypothetical protein	PGN_RS04545	S41 family peptidase
PGN_RS05010	glycoside hydrolase family 2 TIM barrel-domain containing protein	None	None
PGN_RS05100	IS982-like element IS195 family transposase	PGN_RS05105	IS982-like element IS195 family transposase
PGN_RS05875	hypothetical protein	PGN_RS05880	solute carrier family 23 protein
PGN_RS08205	IS982-like element IS195 family transposase	PGN_RS12000	DNA methylase
PGN_RS08420	cation:proton antiporter	PGN_RS08425	Spi family protease inhibitor
PGN_RS09075	S41 family peptidase	PGN_RS09080	hypothetical protein
PGN_RS09260	IS5 family transposase	PGN_RS09265	CRISPR-associated endonuclease Cas2
PGN_RS09660	TolC family protein	PGN_RS09665	DNA mismatch repair protein MutS
PGN_RS11730	hypothetical protein	PGN_RS01530	sigma-70 family RNA polymerase sigma factor

Additionally, several intergenic targets were identified upstream of genes, with a greater emphasis on mobile genetic elements (multiple transposases), protein processing machinery, and transport systems (Table 1). The presence of multiple IS elements suggests that these spacers may target regions involved in genomic instability or horizontal gene transfer events.

Enrichment analysis revealed specific quantitative patterns of spacer targeting, demonstrating a precise regulatory focus on key cellular processes. The most dramatically enriched functional category was selenium compound metabolic process, showing enrichment levels exceeding 100 genes/terms on the percentage scale, followed by substantial enrichment in cobalamin biosynthetic processes and multiple DNA-related processes with varying but significant enrichment levels. However, when analyzing the proportional distribution of targeted functions, tRNA modification emerged as the predominant category, representing 29.41% of all spacer targets, followed by translational elongation at 14.71% (Fig. 4c and d). This indicates that nearly half of all CRISPR spacer targets are focused on the protein synthesis machinery. Additional functional categories showed more modest but significant representation: proteolysis, valyl-tRNA aminoacylation, and regulation of DNA-templated transcription each comprised 8.82% of targets, while selenium compound metabolic process, cobalamin biosynthetic process, and magnesium ion transport each represented 5.88% of the total (Fig. 4c and d). The most specific regulatory targets, DNA transposition, and regulation of DNA replication each accounted for 2.94% of spacer targets. This quantitative distribution reveals that CRISPR array 30.1 spacers predominantly target the translational machinery (tRNA modification and translational elongation combined represent over 44% of targets), suggesting that the primary regulatory mechanism involves fine-tuning protein synthesis capacity and efficiency rather than broad metabolic control, with secondary focus on proteolytic processes and transcriptional regulation.

## DISCUSSION

This study identifies a previously unrecognized regulatory function for the non-coding CRISPR array 30.1 in *P. gingivalis* ATCC 33277, establishing it as a global modulator of bacterial physiology and host-pathogen interactions. Deletion of the CRISPR array 30.1 led to substantial changes in the bacterial transcriptome, including enhanced metabolic and secretion pathways, increased biofilm formation, accelerated virulence in the *G. mellonella* infection model, and a heightened proinflammatory cytokine response in THP-1 macrophages. These findings expand the functional scope of CRISPR–Cas systems beyond nucleic acid-targeted immunity, underscoring their potential as central regulators of bacterial virulence.

While self-targeting spacers are documented across diverse bacterial species, their precise regulatory roles remain largely speculative (5, 10). Our results provide direct evidence of spacer-mediated repression of endogenous genes, as demonstrated by SPA mapping of CRISPR array 30.1 spacers onto self-genomic targets. This regulatory model aligns with mechanisms observed in other bacteria, such as *Francisella novicida*, where Cas9-mediated transcriptional interference, using small CRISPR-associated RNAs (scaRNA), modulates endogenous gene expression (32, 33). In *P. gingivalis*, spacer-target interactions prominently involve genes critical for nutrient acquisition, surface modification, and secretion, central factors in biofilm development and intracellular survival.

Enhanced biofilm formation observed in the ΔCRISPR 30.1 mutant supports the idea that CRISPR array 30.1 negatively regulates biofilm-associated genes, potentially facilitating bacterial transition from biofilm growth to host cell invasion. Biofilms enable the persistence of *P. gingivalis* under dynamic environmental conditions, including fluctuating oxygen and nutrient levels typical of the oral cavity (34, 35). Our findings extend previous reports demonstrating the regulation of novel adhesins essential for virulence in *P. gingivalis* by the type I-B CRISPR–Cas system (25, 26).

Temporal dynamics revealed distinct regulatory phases in the host-pathogen interaction, with bacterial responses showing consistent metabolic upregulation at 2 and 6 h post-*P. gingivalis* infection in THP-1 cells, while host responses demonstrated time-dependent pathway switching. At 2 h, host cells activated growth factor signaling (EGFR inhibitor resistance) and lipid metabolism (via PPAR signaling), suggesting an initial metabolic adaptation to bacterial invasion. *P. gingivalis* is known to manipulate the inflammatory responses of macrophages through signaling pathways, including PPAR activation (36, 37). By 6 h, the response shifted toward carbohydrate metabolism and immune modulation pathways, particularly pertussis-related responses, indicating a transition from metabolic accommodation to active antimicrobial defense. This temporal progression suggests that the CRISPR array 30.1 deletion not only removes metabolic constraints on the bacterium but also disrupts the normal kinetics of host-pathogen equilibrium, leading to accelerated inflammatory activation and loss of the bacterium’s typical immune evasion capabilities.

SPA analysis identified genome-wide self-targeting by CRISPR array 30.1 spacers, including genes and intergenic regions upstream of critical operons. Contrary to expectations based on stress response pathways, tRNA modification emerged as the predominant target category, representing 29.41% of all spacer targets, followed by translational elongation at 14.71%. Collectively, these two categories account for over 44% of the regulatory focus on the protein synthesis machinery. This quantitative distribution directly explains the observed ribosomal pathway enrichments, establishing a clear mechanistic link between spacer targeting and transcriptomic outcomes. Selenium metabolism showed the highest statistical enrichment among targeted pathways, suggesting that CRISPR array 30.1 specifically constrains selenium-dependent processes, which may be critical for anaerobic metabolism and oxidative stress responses in the periodontal environment. The incorporation of selenium into selenoproteins, particularly in the form of selenocysteine, is critical for various physiological functions in bacteria, including redox homeostasis and antioxidant defenses (38).

Notably, we observed spacer-mediated targeting of *cas* loci from type VI-B systems, suggesting auto-regulation of CRISPR immunity and cross-talk between distinct CRISPR-Cas systems. Cross-system interaction has been documented in *Flavobacterium columnare*, where type VI-B systems capture spacers from type II-C systems, acquiring spacers from both phage and host genomic DNA (39). Similarly, type I and III systems in *Serratia* spp. cooperatively share spacers, enhancing overall bacterial immunity (40, 41).

Targets identified in our study also include critical stress response and DNA repair genes, such as UvrA, UvrD, and RecQ helicases. UvrA initiates nucleotide excision repair (NER) by recognizing DNA lesions and recruiting repair complexes (42, 43). UvrD helicase unwinds DNA strands post-damage recognition, facilitating repair, while RecQ helicase contributes to homologous recombination and DNA damage processing (44, 45). These findings imply a conserved role for these proteins in stress management and genomic stability in *P. gingivalis*.

Furthermore, identified spacer targets include genes and operons linked to anaerobic metabolic adaptation, particularly the cobalamin biosynthesis pathway, which is essential under nutrient-stress conditions prevalent during inflammation-associated periodontal disease (46–48). Regulation of cobalamin synthesis pathways by CRISPR could directly influence virulence, particularly through modulation of gingipains, potent virulence factors associated with tissue destruction and immune evasion (49–51). Additionally, CRISPR spacers target regulatory proteins, including AraC family transcriptional regulators. The AraC-like proteins are significant regulatory elements in *Porphyromonas gingivalis*, a prominent pathogen involved in periodontal disease. These proteins play a crucial role in the bacterium’s adaptability and virulence by regulating gene expression in response to environmental signals, particularly those related to surface structures, such as fimbriae, which are critical for *P. gingivalis*’s ability to establish and persist in subgingival biofilm environments. The production of fimbriae in *P. gingivalis* is controlled explicitly by a two-component regulatory system comprising FimS and FimR. Nishikawa et al. detailed how this system positively regulates the expression of the fimA gene, which encodes the major fimbrial protein (52). This system’s functionality is supported by further studies, which indicate that the expression of both long (FimA) and short fimbriae is tightly coordinated through signaling mechanisms involving FimS and FimR (53, 54).

This interaction exemplifies the concept of environmental sensing and transcriptional adaptation, where factors, such as host responses to *P. gingivalis*, signify a dynamic interplay of regulatory actions that govern pathogenicity (55).

Notably, new insights into DNA methylation and gene expression changes triggered by heme availability highlight a complex layer of regulation in *P. gingivalis*. Costeira et al. identified differential DNA methylation signatures that are crucial for understanding how gene expression is modulated by nutritional cues, indicating that methylation processes may be intertwined with regulatory proteins, such as AraC (38). This suggests that AraC family proteins may be part of a broader regulatory network, responding not only to direct environmental stimuli but also to epigenetic changes that influence gene expression. By targeting these regulators, CRISPR arrays likely orchestrate responses to environmental stressors and host immune challenge*. P. gingivalis* is known for its ability to evade innate immunity, suppressing chemokines, such as IL-8, to inhibit neutrophil recruitment and manipulate host inflammatory responses (56–58).

The loss of the CRISPR array 30.1 reversed immune evasion, amplifying THP-1 macrophage secretion of proinflammatory mediators (IL6, IL8, TNFα, CXCL1, CXCL2) and driving a pronounced rise in CXCL9 (~36-fold). CXCL9, an IFNγ–responsive CXCR3 ligand, would be expected to recruit effector T cells and NK cells and bias the response toward a Th1/M1 axis (31, 59, 60), while elevated IL6 together with CXCL1/CXCL2 strengthens an acute neutrophil-rich milieu linked to osteoclastogenesis and bone resorption. The predominance of significance at 6 h is consistent with transcriptional amplification via NFκB/MAPK (for IL6/CXCL1/2) and STAT1/IRF1-responsive programs (for CXCL9) (61, 62). Taken together, the IL6 and CXCL9 signatures indicate that loss of CRISPR array 30.1 heightens macrophage sensing and shifts the host response toward a neutrophil- and CXCR3-mediated inflammatory program. CRISPR array 30.1–dependent modulation of MAPK/ERK and JAK/STAT pathways may intersect with carcinogenic processes reported in epithelial cells infected by *P. gingivalis* (63, 64).

Interestingly, TNF-α had no significant difference between the wild type and the mutant. Further studies dissecting individual spacer-target pairs will be essential to elucidate how CRISPR-mediated regulation of secreted virulence factors influences host-pathogen dynamics.

Our findings complement transposon-sequencing data, which demonstrate essential roles for type I, III, and VI Cas systems in epithelial colonization and *in vivo* abscess formation (21). These results strongly suggest that *P. gingivalis* exploits multiple CRISPR systems, including non-coding arrays, to fine-tune its pathogenic strategies.

This study utilized THP-1 macrophage-like cells and the *G. mellonella* infection model, which, while robust and informative, do not fully replicate human periodontal disease. Although SPA effectively identifies spacer targets, definitive evidence of transcriptional repression requires additional targeted reporter or CRISPR interference (CRISPRi) assays. Future research will focus on testing the interaction between CRISPR array 30.1 and the UvrA gene, due to its essential role in DNA repair and stress response pathways. This targeted approach will inform subsequent experiments and clarify the regulatory mechanisms involved in this process.

The results presented here significantly enhance our understanding of how *P. gingivalis* exploits CRISPR–Cas systems to manipulate the host’s immune response. Notably, the loss of CRISPR array 30.1 led to amplified inflammatory cytokine release and a gene expression profile supporting a shift in macrophage polarization, signaling a heightened proinflammatory response that could disrupt the equilibrium between host defense mechanisms and pathogen persistence. This manipulation of immune signaling is a hallmark of *P. gingivalis*’s pathogenicity (65) and suggests a broader role for CRISPR-mediated regulation in immune modulation. Similar immune-modulatory functions of CRISPR have been reported in other oral pathogens (5). Thus, the *S. mutans* UA159 strain deficient in the *cas*3 gene not only showed decreased EPS production and biofilm formation but also increased fluoride sensitivity (66). Additionally, oral pathogens may utilize CRISPR-Cas to modulate microbe-microbe interactions. *T. forsythia* spacers exhibit significant nucleotide similarity to genes of *P. gingivalis* (e.g., methyltransferase gene) and *T. denticola* (67). The authors suggested that *P. gingivalis* may deliver its DNA into the cells of other species to establish niche dominance, whereas *T. forsythia* attacks the methyltransferase gene of *P. gingivalis* by delivering the spacer and Cas proteins of its *T. forsythia* CRISPR–Cas system into *P. gingivalis* cells to impact their persistence (67).

Future studies should explore individual spacer-target interactions through targeted editing or spacer swapping within CRISPR arrays. Finally, therapeutic approaches, such as small-molecule inhibitors or antisense oligonucleotides disrupting spacer-target interactions, present exciting opportunities for novel antimicrobial strategies. By demonstrating that a non-coding CRISPR array functions as a master regulator of virulence and host interactions, our findings significantly expand the current understanding of CRISPR biology and reveal new avenues for therapeutic intervention against bacterial pathogens.

## MATERIALS AND METHODS

### Bacterial growth conditions

*P. gingivalis* ATCC 33277 and derivative strains were cultured anaerobically at 37°C. TSBH agar plates were used for cell maintenance, supplemented with 5% defibrinated sheep blood, 5 µg/mL hemin, and 1 µg/mL menadione (vitamin K). Liquid cultures were prepared in TSBH supplemented with 20% heat-inactivated human serum, 5 µg/mL hemin, and 1 µg/mL menadione. All reagents were obtained from Fisher.

### Construction of the ΔCRISPR array 30.1 mutant

To construct a CRISPR array 30.1 knockout strain of *P. gingivalis*, we replaced the entire array region with an erythromycin resistance cassette. All plasmids used for construction and confirmation of the ΔCRISPR 30.1 mutant are shown in Table S1.

First, we designed a plasmid (pUC19) carrying the erythromycin resistance cassette (*erm*F gene from pVA2198) flanked by the 1 kb region upstream and downstream of CRISPR array 30.1. The entire CRISPR array 30.1 comprises 119 spacers and repeats, totaling 7.9 Kb in size.

The NEBuilder HiFi DNA assembly kit (New England Biolabs) was used for this construct. Subsequently, the construct (the erythromycin cassette and its flanking regions) was amplified using Pfu polymerase (Fermentas) following the manufacturer’s protocol.

The amplified fragment was purified using an EZNA gel extraction kit (Omega) and used for electroporation of *P. gingivalis* electrocompetent cells. To prepare electrocompetent cells, cells were grown in tryptic soy broth supplemented with hemin and vitamin K to an optical density at 600 nm (OD_600_) of 0.6–0.7. After centrifugation, the cells were washed twice in ice-cold electroporation buffer (10% glycerol, 1 mM MgCl2) and resuspended in a minimal amount of electroporation buffer. Electroporation was performed by adding different amounts of the purified DNA fragment to 100 µL of *P. gingivalis* competent cells. Tryptic soy broth blood agar plates supplemented with hemin, vitamin K, and 10 µg/mL erythromycin were used for the mutant selection. Plates were incubated anaerobically at 37°C for 9 days, and the resulting colonies were streaked on new plates to obtain single colonies. Electroporation conditions were 2.5 kV, 5.0 ms, 400 Ω, and 25 μF. The correct knockout strains were preserved in glycerol and dimethyl sulfoxide (DMSO) stocks and kept in a −80°C freezer.

The successful deletion of the entire spacer repeat array was confirmed using three PCR assays. The primer sets were strategically designed to confirm the presence of the replacement cassette and its correct orientation within the genomic locus (Fig. S5a). All reactions were prepared with a final volume of 50 μL, using 25 μL of Green MM 2×, 1 μL of template DNA, 1 μL of each primer (forward and reverse), and 22 μL of water. Fig. S5b shows that the entire region was indeed removed. A detailed protocol outlining the three PCR conditions is provided in the Jupyter notebook. The amplicons were confirmed by sequencing.

### Biofilm assay

We inoculated 200 μL of bacterial culture (OD₆₀₀ = 0.1) into 96-well polystyrene plates and incubated them anaerobically for 48 h without shaking. After gently washing twice with PBS, the biofilms were stained with 0.1% safranin for 15 min, rinsed with PBS, and air-dried. Bound dye was solubilized in 200 µL 30% acetic acid, and absorbance was measured at 492 nm on a microplate reader.

### *Galleria mellonella* infection model

*G. mellonella* larvae infection model was used in the experiments. Insects in the final instar larval stage were purchased from Vanderhorst, Inc. (St. Marys, OH, USA).

Upon arrival, the larvae were sorted, and any dead larvae were removed. Healthy larvae weighing between 200 and 300 mg without any signs of melanization were selected. Each group consisted of 15 larvae, and seven groups were used for the infection. Infection was performed by injecting 5 µL aliquots of bacterial inoculum into each larva’s hemocoel through the last left proleg using a Hamilton syringe. Three groups received ΔCRISPR array 30.1 mutants, three groups received wild-type *P. gingivalis*, and three control groups were included. The control groups consisted of TSBH plus heat-killed strains (10 min at 75°C) of the ΔCRISPR array 30.1 mutant or *P. gingivalis* wild type; TSBH broth was used as a medium-only control. Inoculated larvae were incubated at 37°C in the dark, and survival was recorded at different time intervals.

Kaplan–Meier survival curves were plotted to assess differences. A *P-*value of ≤0.05 was considered significant. Data analysis was performed using the “*survival*” and “*survminer*” (68) packages in R (see Jupyter notebook for details). Experiments were conducted three times independently, yielding comparative outcomes.

### THP-1 cell culture and infection

Human monocytes THP-1 (ATCC, TIB-202) were grown in a 5% CO_2_ incubator at 37°C in RPMI 1640 and supplemented with L-glutamine (2 mM), heat-inactivated fetal bovine serum (10%), penicillin/streptomycin (100 U/100 μg/mL), sodium pyruvate (1 mM), HEPES (10 mM), glucose (4.5 mg/mL), sodium bicarbonate (1.5 mg/mL), and 2-mercaptoethanol (0.05 mM) (Sigma-Aldrich). The cells were standardized to a concentration of 5 × 10^5^ viable cells/mL. Differentiation into a macrophage-like state was induced by treating the cells with 100 ng/mL phorbol 12-myristate 13-acetate (PMA; Sigma-Aldrich). One milliliter (5 × 10^5^) of THP-1 cells was added to each well of the 24-well cell culture plates. Cells were incubated for 48 h in the presence of PMA, then washed three times with PBS, and the cell medium was replaced with fresh, antibiotic-free medium to receive the bacterial inoculum. There was no resting period with PMA-free medium before bacterial inoculation.

### Bacterial infection experiments

*P. gingivalis* wild-type and ΔCRISPR array 30.1 mutant cells were harvested by centrifugation from BHI broth culture. The bacteria were washed three times in RPMI 1640 medium and adjusted to an optical density (OD_660_) of 1.0 (approximately 1 × 10^9^ CFU/mL). The bacteria were added to PMA-treated THP-1 cells at a multiplicity of infection of 100 in antibiotic-free medium. After 2, 6, and 24 h post-infection, cell culture supernatant fluids were harvested to measure cytokine expression. Cells were then washed twice with PBS, and RNA was extracted for dual transcriptome analysis.

### Cytokine and chemokine quantification

In cell culture supernatants, the levels of TNF-α, IL-1α, IL-6, IL-8, IL-10, and RANTES were determined using Milliplex multiplex assays (EMD, Millipore). Data were obtained using a Luminex 200 system with xPONENT 3.1 software (Luminex) and analyzed with a 5-parameter logistic spline-curve fitting method and Milliplex Analyst V5.1 software (Vigene Tech). Statistical differences were assessed using non-parametric tests for group and time comparisons, specifically the Wilcoxon rank-sum test, followed by a Kruskal-Wallis test with a Dunn’s post-hoc test for multiple comparisons. We applied a false discovery rate (FDR) correction to the *P*-values to account for multiple comparisons (see Jupyter notebook for details). Experiments were performed in triplicate.

### Dual RNAseq and analysis

At 2 and 6 h post-infection, we harvested co-cultures for simultaneous host-pathogen transcriptomics. Cells were washed three times with PBS, and total RNA was extracted using mirVana kits (Thermo Fisher) with 1 min bead beating (0.1 mm diethylpyrocarbonate-treated zirconia-silica beads; BioSpec Products) in lysis buffer. Ribosomal RNA was depleted using RiboZero kits (Illumina), and strand-specific libraries were prepared with TruSeq Stranded Total RNA reagents. Libraries were sequenced on an Illumina NovaSeq platform (2 × 150 bp), yielding >20 million read pairs per sample. Reads were mapped to combined human (GRCh38) and *P. gingivalis* (ASM26048v1) reference genomes with HISAT2, and counts were generated with *featureCounts*. Differential expression was assessed with DESeq2 (|log₂FC| > 0.585, FDR < 0.05), and pathway enrichment was analyzed using the R package *pathfindR (*69*)* (see Jupyter notebook for details).

### Single-primer amplification (SPA)

SPA was based on the universally primed-PCR technique (61). We designed single primers for the 119 spacer sequences, although some of them are repeated in the array, resulting in a total of 109 unique sequences (Table S2). Using ΔCRISPR array 30.1 genomic DNA (to avoid array self-amplification), we performed SPA reactions with each primer-spacer individually (two technical replicates).

SPA was performed in 20 μL reactions using Pfu master mix, with 1 pmol/μL spacer primer and 10 ng total DNA. The PCR reactions included 50 cycles. The first cycle was 7 min at 94°C, 70 s at 55°C, and 60 s at 72°C. The remaining cycles were 50 s at 92°C, 60 s at 55°C, and 50 s at 70°C (extension was prolonged by 2 min in the last step). The PCR products were electrophoresed in 1.7% agarose gels in TBE buffer at 100 V and visualized by SYBR Safe staining solution. Amplicons were cleaned with the EZNA kit (Omega) and sent for sequencing to the Interdisciplinary Center for Biotechnology Research (ICBR) RRID:SCR_019145 at the University of Florida. Amplicons were purified using the ENZA kit (Omega) and sequenced on an Illumina MiSeq (1 × 300 bp). Reads were aligned to the *P. gingivalis* genome with Bowtie2, and peaks of enrichment (≥5 × background) were identified with MACS2 to call spacer binding sites.

## Data Availability

Sequences have been deposited on the BioSample database (https://www.ncbi.nlm.nih.gov/biosample) with BioProject ID PRJNA1357332 and BioSample accessions SAMN53082244–SAMN53082251.

## SUPPLEMENTAL MATERIAL

Figure S1 spectrum.02834-25-s0001.pdf Cytokine and chemokine expression profiles at 2 and 6 h post-treatment across different experimental groups.

Figure S2 spectrum.02834-25-s0002.pdf Principal component analysis (PCA) of *P. gingivalis* and THP‑1 transcriptomes following infection with wild-type or ΔCRISPR 30.1 strains. PCA plots visualize global gene expression profiles of biological replicates for each condition, with 95% confidence ellipses encircling replicates.

Figure S3 spectrum.02834-25-s0003.pdf PathfindR-derived KEGG pathway enrichment during *P. gingivalis* infection of THP‑1 cells.

Figure S4 spectrum.02834-25-s0004.pdf Genome mapping of single‑spacer SPA results in *P. gingivalis* ATCC 33277.

Figure S5 spectrum.02834-25-s0005.pdf Confirmation of the deletion in the mutant ΔCRISPR 30.1.

Supplemental material spectrum.02834-25-s0006.html Jupyter notebook with all bioinformatic code and PCR conditions.

Supplemental legends spectrum.02834-25-s0007.docx Legends for Figures S1 to S5.

Table S1 spectrum.02834-25-s0008.pdf Primers used in the construction and confirmation of the ΔCRISPR-30.1 mutant.

Table S2 spectrum.02834-25-s0009.xlsx Spacers sequences, length, and location in the genome.

ASM does not own the copyrights to Supplemental Material that may be linked to, or accessed through, an article. The authors have granted ASM a non-exclusive, world-wide license to publish the Supplemental Material files. Please contact the corresponding author directly for reuse.
