# Supplementary figures and images for: A CRISPR array orchestrates virulence and host response in Porphyromonas gingivalis
Source: Microbiol Spectr. 2026 Feb 25;14(4):e02834-25. doi: 10.1128/spectrum.02834-25 (PMC13055991; doi:10.1128/spectrum.02834-25)

a)

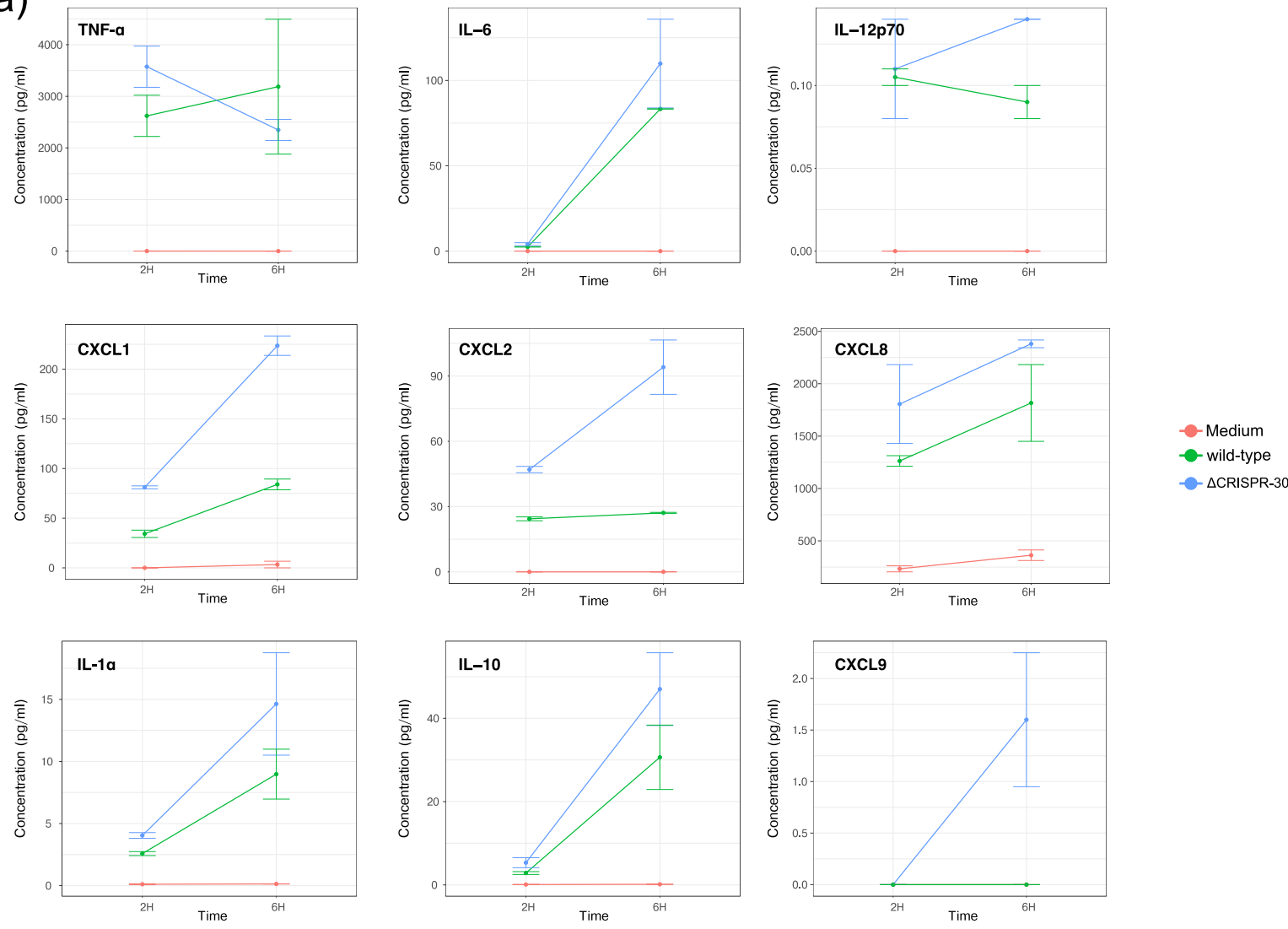

b)

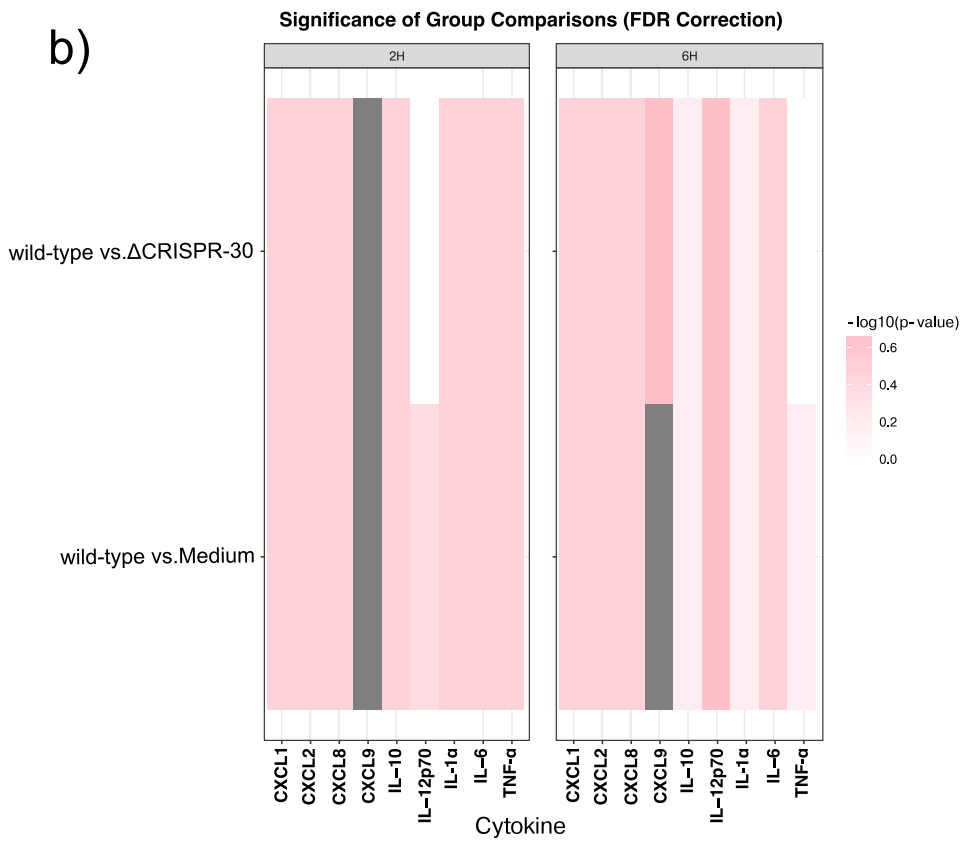

Supplement: Figure S1 — Cytokine and chemokine expression profiles at 2 and 6 h post-treatment across different experimental groups. [file spectrum.02834-25-s0001.pdf]

A)

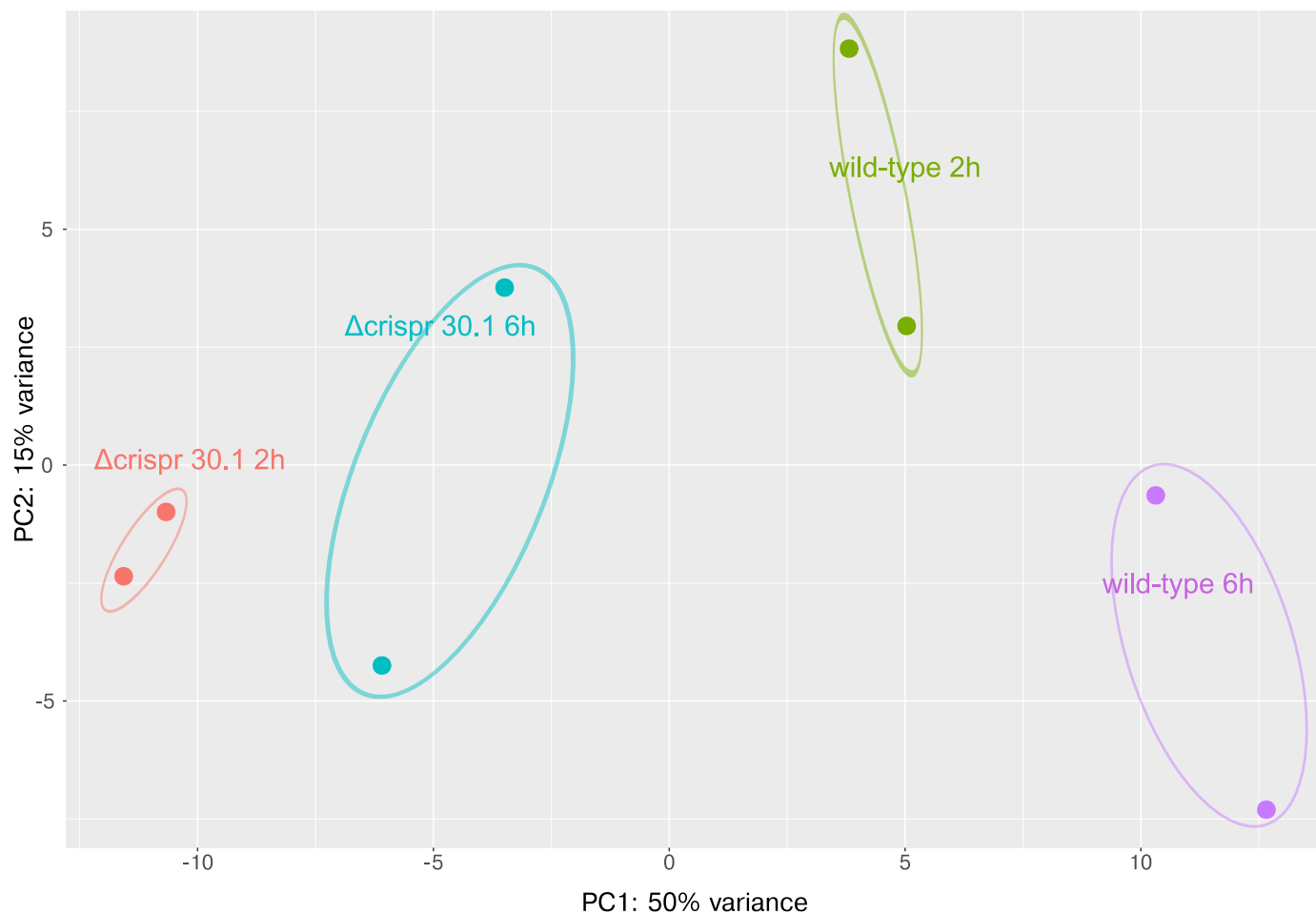

B)

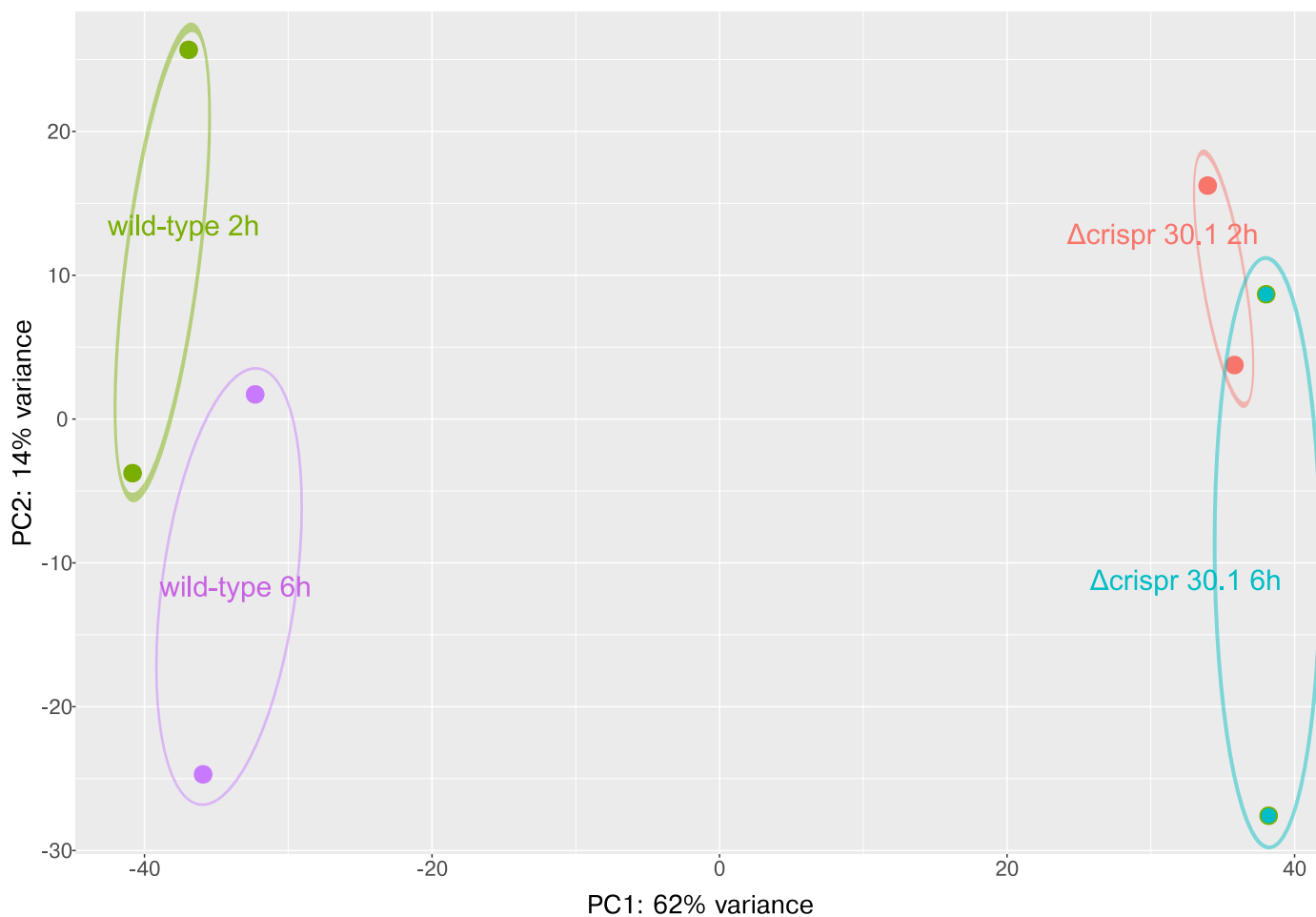

Supplement: Figure S2 — Principal component analysis (PCA) of P. gingivalis and THP‑1 transcriptomes following infection with wild-type or ΔCRISPR 30.1 strains. PCA plots visualize global gene expression profiles of biological replicates for each condition, with 95% confidence ellipses encircling replicates. [file spectrum.02834-25-s0002.pdf]

A)

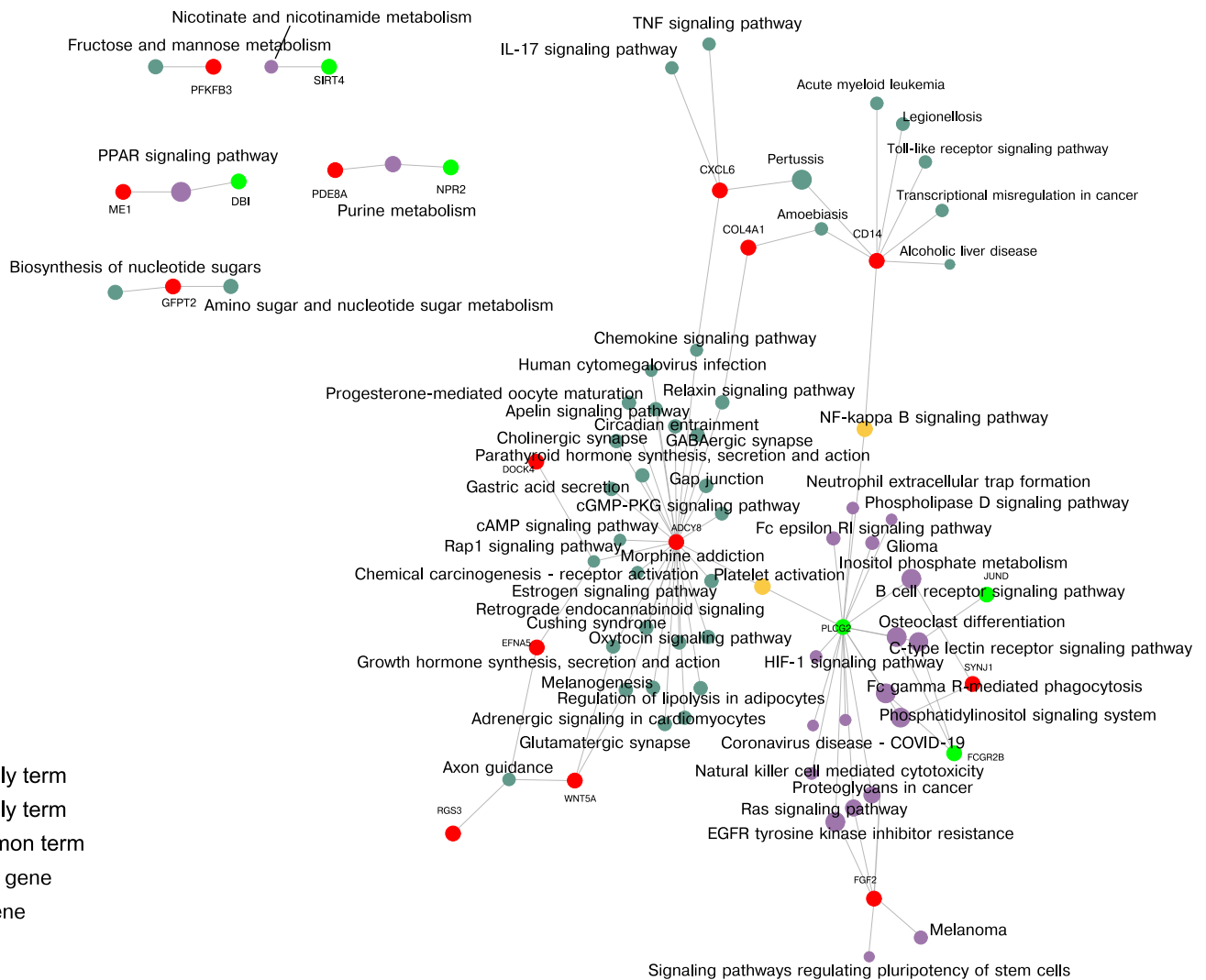

B)

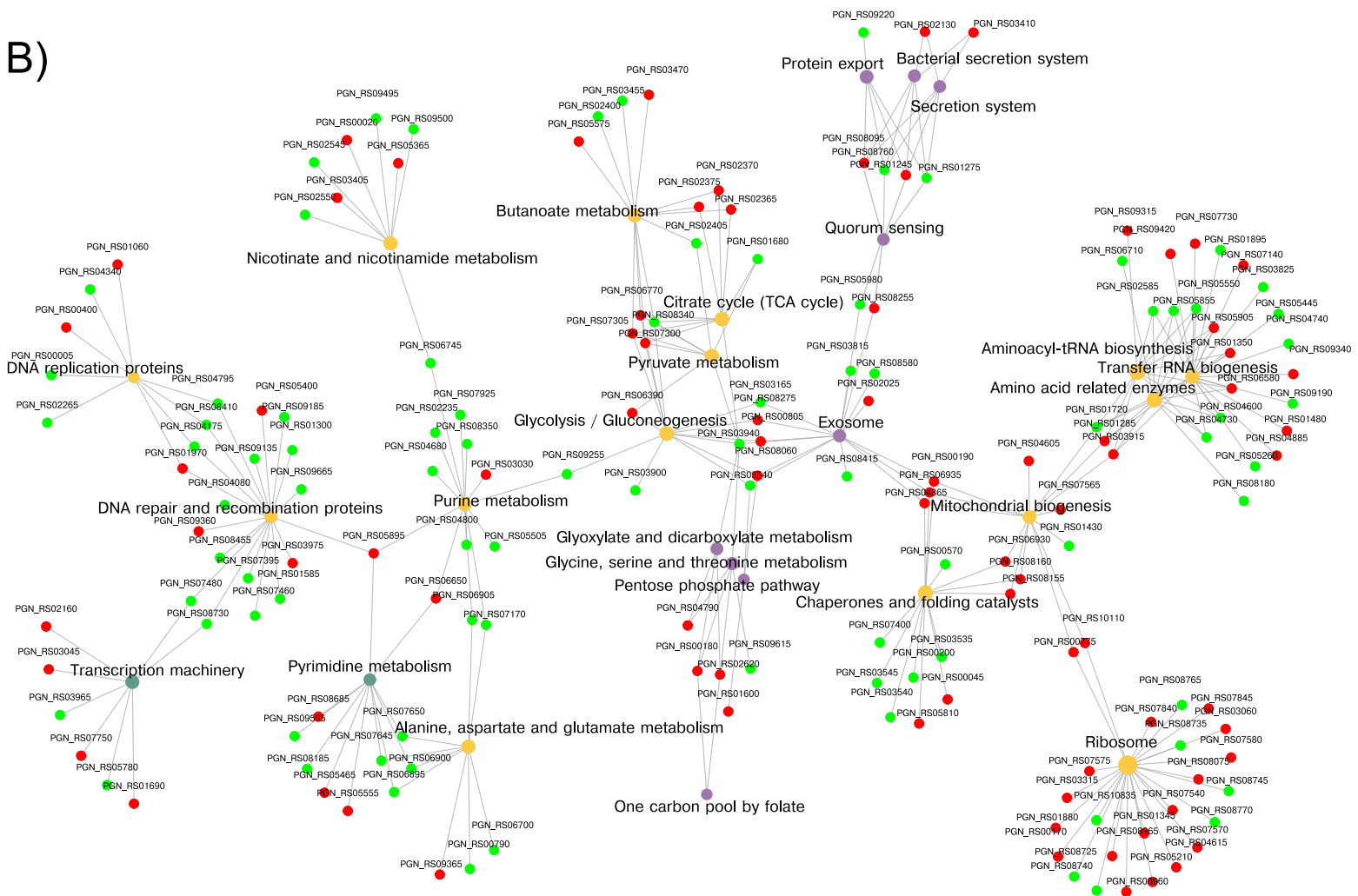

Supplement: Figure S3 — PathfindR-derived KEGG pathway enrichment during P. gingivalis infection of THP‑1 cells. [file spectrum.02834-25-s0003.pdf]

A)

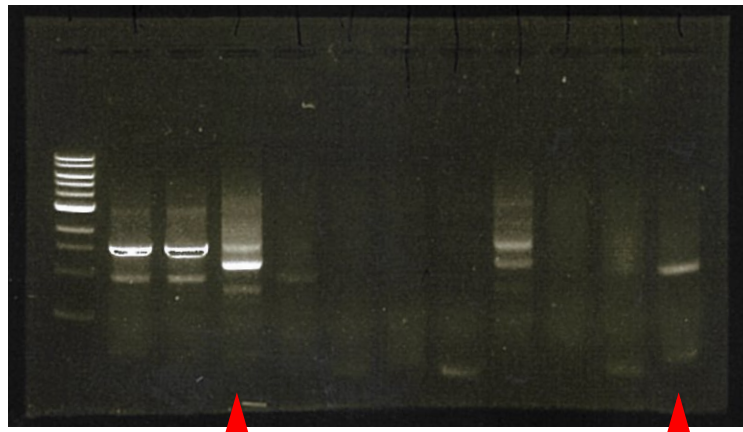

CRISPR\_30.1-10

CRISPR\_30.1-2

B)

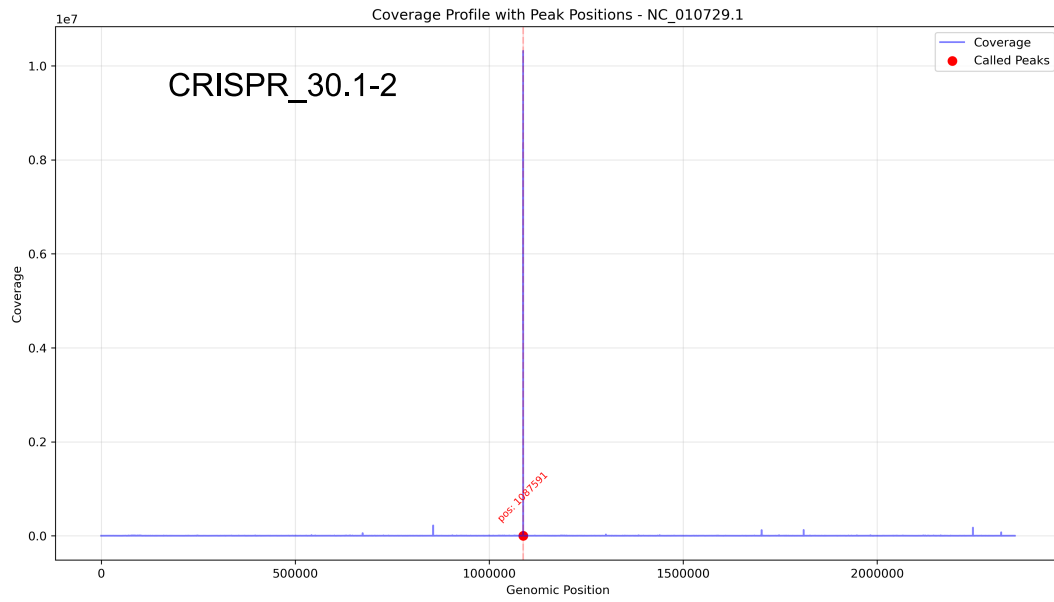

C)

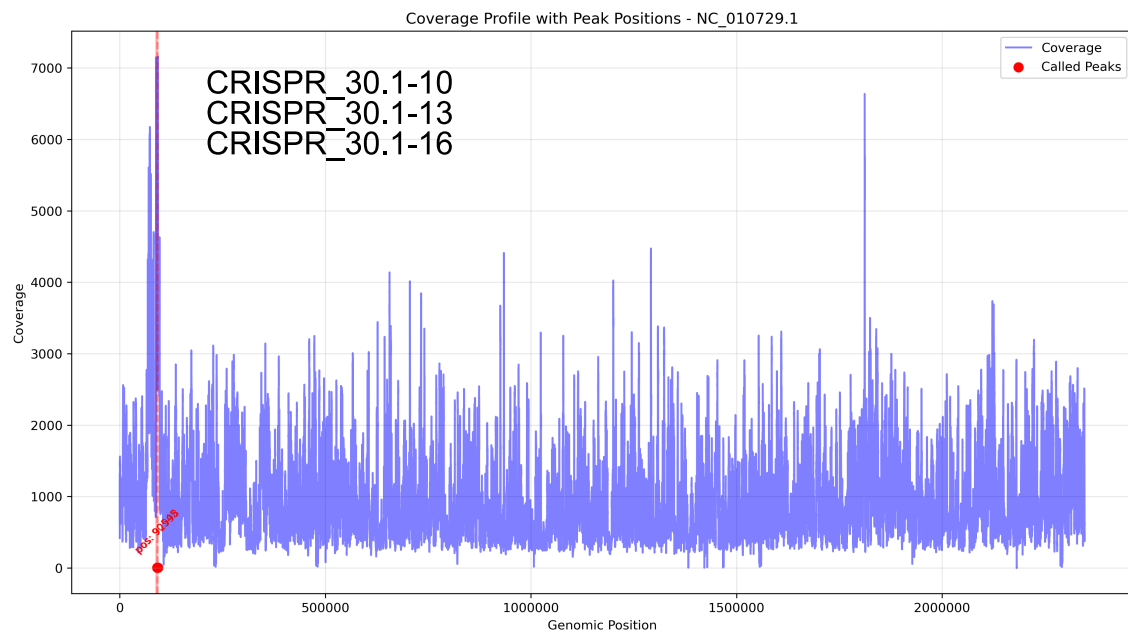

Supplement: Figure S4 — Genome mapping of single‑spacer SPA results in P. gingivalis ATCC 33277. [file spectrum.02834-25-s0004.pdf]
