# Supplementary material for: A CRISPR array orchestrates virulence and host response in Porphyromonas gingivalis
Source: Microbiol Spectr. 2026 Feb 25;14(4):e02834-25. doi: 10.1128/spectrum.02834-25 (PMC13055991; doi:10.1128/spectrum.02834-25)
Supplement: Figure S5 — Confirmation of the deletion in the mutant ΔCRISPR 30.1. [file spectrum.02834-25-s0005.pdf]

A)

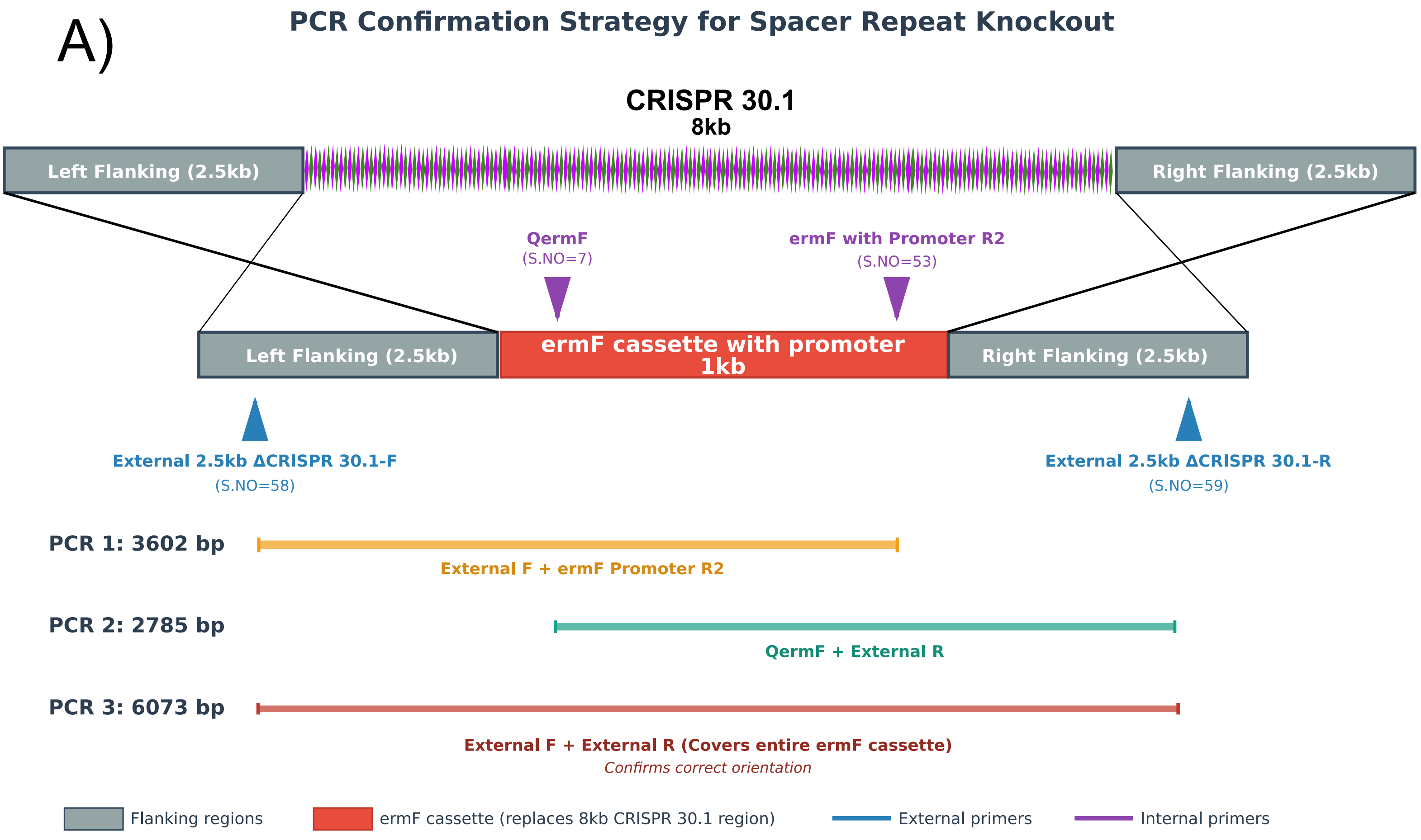

B)

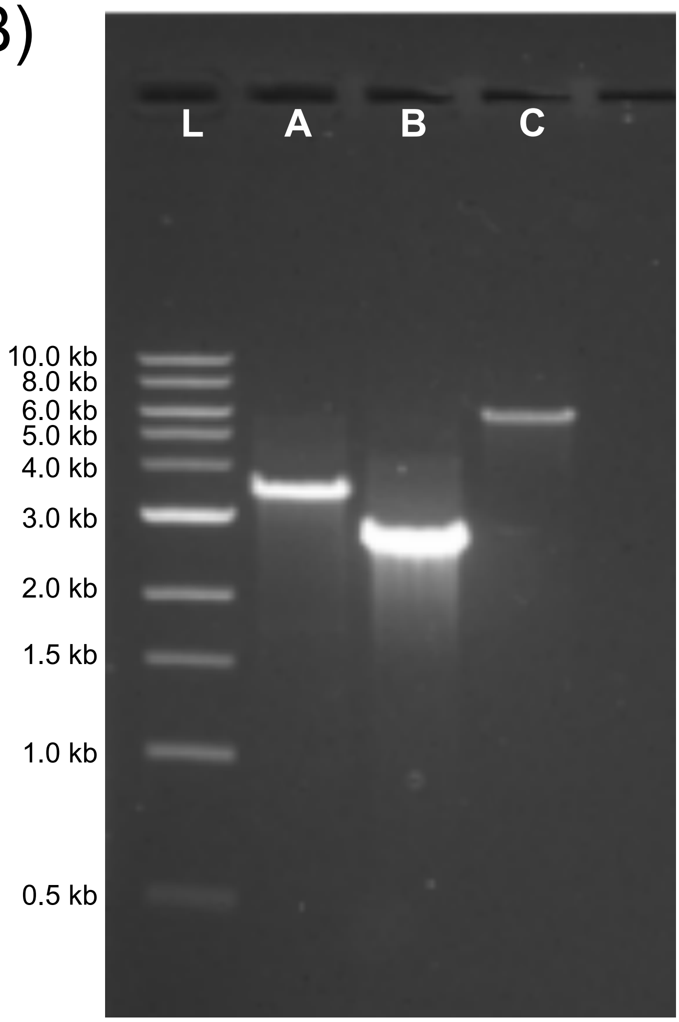

(L) Quick load 1 kb DNA ladder (NEB)
(A) PCR 1 Product size 3602 bp
(B) PCR 2 Product size 2785 bp
(C) PCR 3 Product size 6073 bp
