# Supplementary material for: A CRISPR array orchestrates virulence and host response in Porphyromonas gingivalis
Source: Microbiol Spectr. 2026 Feb 25;14(4):e02834-25. doi: 10.1128/spectrum.02834-25 (PMC13055991; doi:10.1128/spectrum.02834-25)
Supplement: Supplemental material — Jupyter notebook with all bioinformatic code and PCR conditions. [file spectrum.02834-25-s0006.html]

Irfan\_et\_al\_notebook


In [ ]:

```
############################# Figure 1c,d and e. Kaplan-Meier survival curves #################################################
### In R

library(survival)
library(survminer)
library(dplyr)

mydata<-read.table('mydata.txt', header = TRUE)
surv_object <- Surv(time = mydata$time, event = mydata$status)
fit1 <- survfit(surv_object ~ group, data = mydata)
ggsurvplot(fit1, data = mydata, pval = TRUE)


######### data has teh following format:

group	time	status
TSB/Media	0	0
TSB/Media	0	0
TSB/Media	0	0
TSB/Media	0	0

...
```

In [ ]:

```
################################## Figure 2 and Figure S1. Cytokine/chemokine analysis #################################################

# Load required libraries
library(ggplot2)
library(dplyr)
library(tidyr)
library(purrr)
library(rstatix)
library(gridExtra)

# Read the data
df <- read.table("boxplot_all_no-LPS.txt", header=TRUE)

# Extract time and group information from the Label column
df <- df %>%
  mutate(
    time = sub("^([^_]+)_.*$", "\\1", Label),
    group = sub("^[^_]+_(.*)$", "\\1", Label)
  )

# Filter to include only 2H and 6H timepoints
df <- df %>% filter(time %in% c("2H", "6H"))

# Function to perform statistical comparison between wild-type and other groups
compare_groups <- function(data, timepoint) {
  # Filter data for the specific timepoint
  df_time <- data %>% filter(time == timepoint)
  
  # Identify the wild-type group
  wt_group <- "wt"
  
  # Get unique cytokines
  cytokines <- unique(df_time$variable)
  
  # Get unique groups excluding wild-type
  other_groups <- unique(df_time$group)
  other_groups <- other_groups[other_groups != wt_group]
  
  # Initialize results dataframe
  results <- data.frame(
    Timepoint = character(),
    Cytokine = character(),
    Comparison = character(),
    p_value = numeric(),
    Significant = character(),
    stringsAsFactors = FALSE
  )
  
  # Loop through each cytokine
  for (cytokine in cytokines) {
    # Filter data for the specific cytokine
    df_cytokine <- df_time %>% filter(variable == cytokine)
    
    # Loop through each other group
    for (other_group in other_groups) {
      # Extract wild-type and other group data
      wt_data <- df_cytokine %>% filter(group == wt_group) %>% pull(value)
      other_data <- df_cytokine %>% filter(group == other_group) %>% pull(value)
      
      # Skip if not enough data points
      if (length(wt_data) < 2 || length(other_data) < 2) {
        next
      }
      
      # Perform Wilcoxon rank-sum test (non-parametric alternative to t-test)
      test_result <- wilcox.test(wt_data, other_data)
      
      # Add result to results dataframe
      results <- rbind(results, data.frame(
        Timepoint = timepoint,
        Cytokine = cytokine,
        Comparison = paste(wt_group, "vs", other_group),
        p_value = test_result$p.value,
        Significant = ifelse(test_result$p.value < 0.05, "Yes", "No"),
        stringsAsFactors = FALSE
      ))
    }
  }
  
  return(results)
}

# Function to compare timepoints within groups
compare_timepoints <- function(data, group) {
  # Filter data for the specific group
  df_group <- data %>% filter(group == group)
  
  # Get unique cytokines
  cytokines <- unique(df_group$variable)
  
  # Initialize results dataframe
  results <- data.frame(
    Group = character(),
    Cytokine = character(),
    Comparison = character(),
    p_value = numeric(),
    Significant = character(),
    stringsAsFactors = FALSE
  )
  
  # Loop through each cytokine
  for (cytokine in cytokines) {
    # Filter data for the specific cytokine
    df_cytokine <- df_group %>% filter(variable == cytokine)
    
    # Extract 2H and 6H data
    data_2h <- df_cytokine %>% filter(time == "2H") %>% pull(value)
    data_6h <- df_cytokine %>% filter(time == "6H") %>% pull(value)
    
    # Skip if not enough data points
    if (length(data_2h) < 2 || length(data_6h) < 2) {
      next
    }
    
    # Perform Wilcoxon rank-sum test
    test_result <- wilcox.test(data_2h, data_6h)
    
    # Add result to results dataframe
    results <- rbind(results, data.frame(
      Group = group,
      Cytokine = cytokine,
      Comparison = "2H vs 6H",
      p_value = test_result$p.value,
      Significant = ifelse(test_result$p.value < 0.05, "Yes", "No"),
      stringsAsFactors = FALSE
    ))
  }
  
  return(results)
}

# Run comparisons for each timepoint with FDR correction
# First create a function that applies FDR correction
compare_groups_with_correction <- function(data, timepoint) {
  # First run the regular analysis
  results <- compare_groups(data, timepoint)
  
  # Get unique cytokines
  cytokines <- unique(results$Cytokine)
  
  # Apply FDR correction within each cytokine
  corrected_results <- data.frame()
  for (cyt in cytokines) {
    # Filter results for this cytokine
    cyt_results <- results %>% filter(Cytokine == cyt)
    
    # Apply FDR correction using Benjamini-Hochberg method
    cyt_results$p_adj <- p.adjust(cyt_results$p_value, method = "fdr")
    cyt_results$Significant <- ifelse(cyt_results$p_adj < 0.05, "Yes", "No")
    
    # Add to corrected results
    corrected_results <- rbind(corrected_results, cyt_results)
  }
  
  return(corrected_results)
}

# Run comparisons for each timepoint with BH correction
results_2h <- compare_groups_with_correction(df, "2H")
results_6h <- compare_groups_with_correction(df, "6H")

# Combine results
results_group_comparisons <- rbind(results_2h, results_6h)

# Sort by significance and p-value
results_group_comparisons <- results_group_comparisons %>%
  arrange(Timepoint, Cytokine, p_adj)  # Now sorting by adjusted p-value

# Apply FDR correction to timepoint comparisons
compare_timepoints_with_correction <- function(data, group) {
  # First run the regular analysis
  results <- compare_timepoints(data, group)
  
  # Apply FDR correction using Benjamini-Hochberg method
  results$p_adj <- p.adjust(results$p_value, method = "fdr")
  results$Significant <- ifelse(results$p_adj < 0.05, "Yes", "No")
  
  return(results)
}

# Run timepoint comparisons for each group with BH correction
results_timepoint <- lapply(groups, function(g) {
  compare_timepoints_with_correction(df, g)
}) %>% bind_rows()

# Sort by significance and adjusted p-value
results_timepoint <- results_timepoint %>%
  arrange(Group, p_adj)

# Print timepoint comparison results
cat("\nTimepoint Comparison Results (2H vs 6H):\n")
print(results_timepoint)

# Perform multiple comparisons using Kruskal-Wallis and Dunn's test
perform_multiple_comparisons <- function(data, cytokine_name) {
  # Filter data for the specific cytokine
  cytokine_data <- data %>% filter(variable == cytokine_name)
  
  # Convert time and group to factors
  cytokine_data$time <- factor(cytokine_data$time)
  cytokine_data$group <- factor(cytokine_data$group)
  
  # Create a combined factor for time and group
  cytokine_data$time_group <- factor(paste(cytokine_data$time, cytokine_data$group, sep = "_"))
  
  # Perform Kruskal-Wallis test
  kw_test <- kruskal.test(value ~ time_group, data = cytokine_data)
  
  if (kw_test$p.value < 0.05) {
    # Significant differences exist, perform Dunn's test with FDR correction
    dunn_test <- cytokine_data %>%
      dunn_test(value ~ time_group, p.adjust.method = "fdr")  # Changed to "fdr"
    
    return(list(
      cytokine = cytokine_name,
      kruskal_p = kw_test$p.value,
      dunn_results = dunn_test
    ))
  } else {
    return(list(
      cytokine = cytokine_name,
      kruskal_p = kw_test$p.value,
      dunn_results = NULL
    ))
  }
}

# Calculate summary statistics for each group, time, and cytokine
summary_stats <- df %>%
  group_by(group, time, variable) %>%
  summarize(
    n = n(),
    mean = mean(value, na.rm = TRUE),
    sd = sd(value, na.rm = TRUE),
    median = median(value, na.rm = TRUE),
    min = min(value, na.rm = TRUE),
    max = max(value, na.rm = TRUE),
    .groups = "drop"
  )

# Print summary statistics
cat("\nSummary Statistics:\n")
print(summary_stats)

# Save results to files
write.csv(results_group_comparisons %>% 
            select(Timepoint, Cytokine, Comparison, p_value, p_adj, Significant), 
          "group_comparison_results.csv", row.names = FALSE)

write.csv(results_timepoint %>% 
            select(Group, Cytokine, Comparison, p_value, p_adj, Significant), 
          "timepoint_comparison_results.csv", row.names = FALSE)

write.csv(summary_stats, "summary_statistics.csv", row.names = FALSE)

#----------------------------------------------------
# Comprehensive Plotting Section
#----------------------------------------------------

# Function to create advanced boxplots
create_advanced_boxplot <- function(data, cytokine_name) {
  # Filter data for the specific cytokine
  cytokine_data <- data %>% filter(variable == cytokine_name)
  
  # Convert time to a factor with levels in correct order
  cytokine_data$time <- factor(cytokine_data$time, levels = c("2H", "6H"))
  
  # Create the plot
  p <- ggplot(cytokine_data, aes(x = group, y = value, fill = time)) +
    geom_boxplot(position = position_dodge(width = 0.85), width = 0.7, alpha = 0.7) +
    geom_point(aes(color = time), position = position_dodge(width = 0.85), size = 2, alpha = 0.7) +
    labs(title = paste("Cytokine:", cytokine_name),
         x = "Group", y = "Concentration") +
    theme_bw() +
    theme(
      axis.text.x = element_text(angle = 45, hjust = 1),
      plot.title = element_text(size = 14, face = "bold"),
      legend.title = element_text(size = 12),
      legend.text = element_text(size = 10),
      axis.title = element_text(size = 12),
      axis.text = element_text(size = 10)
    ) +
    scale_fill_brewer(palette = "Set1") +
    scale_color_brewer(palette = "Set1")
  
  return(p)
}

# Function to create a faceted plot by timepoint for each cytokine
create_faceted_plot <- function(data, cytokine_name) {
  # Filter data for the specific cytokine
  cytokine_data <- data %>% filter(variable == cytokine_name)
  
  # Convert time to a factor with levels in correct order
  cytokine_data$time <- factor(cytokine_data$time, levels = c("2H", "6H"))
  
  # Create a faceted plot
  p <- ggplot(cytokine_data, aes(x = group, y = value, fill = group)) +
    geom_boxplot(alpha = 0.7, outlier.shape = NA) +
    geom_point(position = position_jitter(width = 0.2, height = 0), 
               size = 2, alpha = 0.7, aes(color = group)) +
    facet_wrap(~ time, scales = "free_y", ncol = 2) +
    labs(title = paste("Cytokine:", cytokine_name),
         x = "Group", y = "Concentration") +
    theme_bw() +
    theme(
      axis.text.x = element_text(angle = 45, hjust = 1),
      plot.title = element_text(size = 14, face = "bold"),
      strip.background = element_rect(fill = "lightgray"),
      strip.text = element_text(size = 12, face = "bold"),
      legend.position = "right"
    )
  
  return(p)
}

# Function to create a plot showing time progression for each group
create_time_progression_plot <- function(data, cytokine_name) {
  # Filter data for the specific cytokine
  cytokine_data <- data %>% filter(variable == cytokine_name)
  
  # Calculate mean and standard error for each group and time
  summary_data <- cytokine_data %>%
    group_by(group, time) %>%
    summarize(
      mean_value = mean(value, na.rm = TRUE),
      se = sd(value, na.rm = TRUE) / sqrt(n()),
      .groups = "drop"
    )
  
  # Convert time to a factor with levels in correct order
  summary_data$time <- factor(summary_data$time, levels = c("2H", "6H"))
  
  # Create a line plot showing time progression
  p <- ggplot(summary_data, aes(x = time, y = mean_value, group = group, color = group)) +
    geom_line(size = 1) +
    geom_point(size = 3) +
    geom_errorbar(aes(ymin = mean_value - se, ymax = mean_value + se), width = 0.2) +
    labs(title = paste("Time Progression of", cytokine_name),
         x = "Time", y = paste(cytokine_name, "Concentration (Mean ± SE)")) +
    theme_bw() +
    theme(
      plot.title = element_text(size = 14, face = "bold"),
      legend.title = element_text(size = 12),
      legend.text = element_text(size = 10),
      axis.title = element_text(size = 12),
      axis.text = element_text(size = 10)
    )
  
  return(p)
}

# Function to create bar plots showing fold changes relative to wt
create_fold_change_plot <- function(data, cytokine_name) {
  # Filter data for the specific cytokine
  cytokine_data <- data %>% filter(variable == cytokine_name)
  
  # Calculate mean values for each group and time
  summary_data <- cytokine_data %>%
    group_by(group, time) %>%
    summarize(
      mean_value = mean(value, na.rm = TRUE),
      .groups = "drop"
    )
  
  # Define the wild-type group
  wt_group <- "wt"
  
  # Get wild-type values for each timepoint
  wt_values <- summary_data %>%
    filter(group == wt_group) %>%
    select(time, wt_value = mean_value)
  
  # Calculate fold change relative to wild-type
  fold_change_data <- summary_data %>%
    left_join(wt_values, by = "time") %>%
    mutate(
      fold_change = ifelse(wt_value == 0, NA, mean_value / wt_value),
      fold_change = ifelse(is.infinite(fold_change) | is.nan(fold_change), NA, fold_change)
    ) %>%
    filter(group != wt_group) # Exclude wt group from plot
  
  # Convert time to a factor with levels in correct order
  fold_change_data$time <- factor(fold_change_data$time, levels = c("2H", "6H"))
  
  # Create a bar plot of fold changes
  p <- ggplot(fold_change_data, 
              aes(x = group, y = fold_change, fill = time)) +
    geom_bar(stat = "identity", position = position_dodge(width = 0.9), width = 0.8) +
    geom_hline(yintercept = 1, linetype = "dashed", color = "black") +
    labs(title = paste("Fold Change Relative to WT for", cytokine_name),
         x = "Group", y = "Fold Change") +
    theme_bw() +
    theme(
      axis.text.x = element_text(angle = 45, hjust = 1),
      plot.title = element_text(size = 14, face = "bold")
    ) +
    scale_fill_brewer(palette = "Set1")
  
  return(p)
}

# Function to add statistical significance markers to plots
add_stat_annotation <- function(plot, group_results, timepoint_results, cytokine_name) {
  # Filter results for the specific cytokine
  group_sig <- group_results %>% 
    filter(Cytokine == cytokine_name, Significant == "Yes")
  
  time_sig <- timepoint_results %>% 
    filter(Cytokine == cytokine_name, Significant == "Yes")
  
  # Add significance annotations if needed
  if(nrow(group_sig) > 0 || nrow(time_sig) > 0) {
    # Add significance annotations
    plot <- plot + 
      labs(subtitle = paste("* indicates significant difference (p < 0.05, FDR-corrected)"))
  }
  
  return(plot)
}

# Function to create a significance heatmap for group comparisons
create_group_heatmap <- function(group_results) {
  # Prepare data for heatmap
  heatmap_data <- group_results %>%
    mutate(
      log_p = -log10(p_adj)  # Using FDR-adjusted p-values
    )
  
  # Create heatmap
  p <- ggplot(heatmap_data, aes(x = Cytokine, y = Comparison, fill = log_p)) +
    geom_tile() +
    scale_fill_gradient2(
      low = "white", 
      high = "red", 
      mid = "pink",
      midpoint = -log10(0.05)/2,
      name = "-log10(p-value)"
    ) +
    geom_text(aes(label = ifelse(Significant == "Yes", "*", "")), 
              color = "black", size = 4) +
    labs(title = "Significance of Group Comparisons (FDR Correction)",
         x = "Cytokine", y = "Comparison") +
    theme_bw() +
    theme(
      axis.text.x = element_text(angle = 90, hjust = 1, vjust = 0.5),
      plot.title = element_text(size = 14, face = "bold")
    ) +
    facet_wrap(~ Timepoint, scales = "free_y")
  
  return(p)
}

# Function to create a heatmap for timepoint comparisons
create_timepoint_heatmap <- function(timepoint_results) {
  # Prepare data for heatmap
  heatmap_data <- timepoint_results %>%
    mutate(
      log_p = -log10(p_adj)  # Using FDR-adjusted p-values
    )
  
  # Create heatmap
  p <- ggplot(heatmap_data, aes(x = Cytokine, y = Group, fill = log_p)) +
    geom_tile() +
    scale_fill_gradient2(
      low = "white", 
      high = "blue",
      mid = "lightblue",
      midpoint = -log10(0.05)/2,
      name = "-log10(p-value)"
    ) +
    geom_text(aes(label = ifelse(Significant == "Yes", "*", "")), 
              color = "black", size = 4) +
    labs(title = "Significance of 2H vs 6H Comparisons (FDR Correction)",
         x = "Cytokine", y = "Group") +
    theme_bw() +
    theme(
      axis.text.x = element_text(angle = 90, hjust = 1, vjust = 0.5),
      plot.title = element_text(size = 14, face = "bold")
    )
  
  return(p)
}

# Get unique cytokines
cytokines <- unique(df$variable)

# Create a list to store all plots
all_plots <- list()

# Create plots for each cytokine
for (cyt in cytokines) {
  # Create basic boxplot
  p1 <- create_advanced_boxplot(df, cyt)
  
  # Add statistical annotations
  p1 <- add_stat_annotation(p1, results_group_comparisons, results_timepoint, cyt)
  
  # Create faceted plot by timepoint
  p2 <- create_faceted_plot(df, cyt)
  
  # Create time progression plot
  p3 <- create_time_progression_plot(df, cyt)
  
  # Create fold change plot (if possible)
  p4 <- tryCatch({
    create_fold_change_plot(df, cyt)
  }, error = function(e) {
    # Return an empty plot with error message if fold change calculation fails
    ggplot() + 
      annotate("text", x = 0.5, y = 0.5, 
               label = paste("Cannot calculate fold change for", cyt, "\n", e$message)) +
      theme_void()
  })
  
  # Store plots in the list
  all_plots[[paste0(cyt, "_boxplot")]] <- p1
  all_plots[[paste0(cyt, "_faceted")]] <- p2
  all_plots[[paste0(cyt, "_progression")]] <- p3
  all_plots[[paste0(cyt, "_fold_change")]] <- p4
}

# Create significance heatmaps
p_heatmap_group <- create_group_heatmap(results_group_comparisons)
p_heatmap_timepoint <- create_timepoint_heatmap(results_timepoint)

all_plots[["heatmap_group"]] <- p_heatmap_group
all_plots[["heatmap_timepoint"]] <- p_heatmap_timepoint

# Save all plots to PDF
pdf("cytokine_analysis_plots.pdf", width = 10, height = 8)
for (i in 1:length(all_plots)) {
  print(all_plots[[i]])
}
dev.off()

# Create a combined figure for each cytokine
for (cyt in cytokines) {
  # Arrange plots in a grid
  combined_plot <- grid.arrange(
    all_plots[[paste0(cyt, "_boxplot")]],
    all_plots[[paste0(cyt, "_faceted")]],
    all_plots[[paste0(cyt, "_progression")]],
    all_plots[[paste0(cyt, "_fold_change")]],
    ncol = 2,
    top = paste("Analysis of", cyt)
  )
  
  # Save the combined plot
  ggsave(paste0("combined_", cyt, ".pdf"), combined_plot, width = 12, height = 10)
}

# Create a summary plot with heatmaps
summary_plot <- grid.arrange(
  p_heatmap_group,
  p_heatmap_timepoint,
  ncol = 1
)
ggsave("significance_summary.pdf", summary_plot, width = 12, height = 12)

# Print a summary of findings
cat("\nSummary of Significant Findings (with FDR correction):\n")

# Group comparisons
sig_group_results <- results_group_comparisons %>% 
  filter(Significant == "Yes") %>%
  arrange(Timepoint, Cytokine)

cat("\nSignificant Group Differences:\n")
print(sig_group_results[, c("Timepoint", "Cytokine", "Comparison", "p_value", "p_adj")])

# Timepoint comparisons
sig_time_results <- results_timepoint %>% 
  filter(Significant == "Yes") %>%
  arrange(Group, Cytokine)

cat("\nSignificant Changes Between 2H and 6H:\n")
print(sig_time_results[, c("Group", "Cytokine", "p_value", "p_adj")])
```

In [ ]:

```
#################################################################################################################################
##### Figure 3 and Figure S2.                TRANSCRIPTOME ANALYSIS
## In R

###################################### Sequence cleaning and aligment with STAR #################################################

TrimmomaticPE -threads 35 -phred33 sample_R1.fastq.gz sample_R2.fastq.gz sample_R1_paired.fastq.gz sample_R1_unpaired.fastq.gz sample_R2_paired.fastq.gz sample_R2_unpaired.fastq.gz LEADING:3 TRAILING:3 SLIDINGWINDOW:4:15 MINLEN:36

## Microbiome database generation
STAR --runMode genomeGenerate --runThreadN 35 --genomeDir STAR_microbiome/ --genomeFastaFiles Porphyromonas_gingivalis_genome.fasta --genomeChrBinNbits 12 --limitGenomeGenerateRAM 10537511861

##  Running STAR for microbiome analysis
STAR --genomeDir STAR_microbiome/ --runThreadN 35 --readFilesIn sample_R1_paired.fastq.gz sample_R2_paired.fastq.gz --outSAMtype BAM Unsorted --outFilterMultimapNmax 35 --outFilterScoreMinOverLread 0.50 --alignIntronMax 1 --genomeLoad LoadAndKeep --readFilesCommand zcat --outFileNamePrefix sample-microbiome

## Human database generation
STAR --runMode genomeGenerate --runThreadN 35 --genomeDir STAR_human/ --genomeFastaFiles STAR_human/GCF_000001405.40_GRCh38.p14_genomic.fna --sjdbGTFfile STAR_human/GCF_000001405.40_GRCh38.p14_genomic.gff

## Running STAR for host analysis
STAR --genomeDir STAR_human/ --runThreadN 35 --limitBAMsortRAM 56520374542 --readFilesIn sample_R1_paired.fastq.gz sample_R2_paired.fastq.gz --seedSearchStartLmax 25 --outSAMtype BAM SortedByCoordinate --outSAMattributes NH HI AS NM MD --outFilterType BySJout --outFilterMismatchNmax 2 --outFilterMultimapNmax 20 --outFilterMismatchNoverReadLmax 0.04 --alignIntronMin 20 --alignSJoverhangMin 8 --alignSJDBoverhangMin 1 --sjdbScore 1 --genomeLoad LoadAndKeep --outSAMstrandField intronMotif --readFilesCommand zcat --outFileNamePrefix sample

## Counts microbiome
featureCounts -B -M -p -O -T 35 -t gene -g gene_id -a Porphyromonas_gingivalis_genome.gff -o gene_counts_stable_vs_progressing.txt sample.bam

# Counts human
featureCounts -B -M -p -O -T 35 -t gene -g gene -a GCF_000001405.40_GRCh38.p14_genomic.gff -o gene_counts_stable_vs_progressing.txt sample.bam
```

In [ ]:

```
############# Differential expression analysis
### In R

library(DESeq2)
library(apeglm)
library(BiocParallel)
register(MulticoreParam(30))
library("genefilter")
library(ggrepel)

countData <- read.table('gene_counts.tab', header=TRUE, row.names = 1)
countData=countData[which(rowSums(countData) > 0),]

colData<-data.frame(row.names=colnames(countData),condition=as.factor(c("Negative", "Negative","Negative","Positive","Positive","Positive")))


dds <- DESeqDataSetFromMatrix(countData = countData,colData = colData,design = ~ condition)
keep <- rowSums(counts(dds)) >= 10
dds <- dds[keep,]

dds <- DESeq(dds, fitType='local')
res <- results(dds, contrast=c("condition","WT","KO"))
summary(res)

############ We can order our results table by the smallest adjusted p value: ################################################

sum(res$padj < 0.05 , na.rm=TRUE) ### How many adjusted p-values were less than 0.05?

resOrdered <- res[order(res$padj),]

## FILTER SIGNIFICANT GENES ACCORDING TO SOME CHOSEN THRESHOLD FOR THE FALSE DISCOVERY RATE (FDR) in this case 0.05

resSig <- subset(resOrdered, padj < 0.05)

write.table(resSig, file="DESeq_Results_FDR005.txt")  # Only significant genes

plotMA(res, main="DESeq2", ylim=c(-2,2))


# Genes upregulated in WT (log2FoldChange > 1 & padj < 0.05)

up_in_WT <- res[which(res$padj < 0.05 & res$log2FoldChange > 1), ]
write.csv(as.data.frame(up_in_WT), file = "upregulated_in_WT.csv")

# Genes upregulated in KO (log2FoldChange < -1 & padj < 0.05)

up_in_KO <- res[which(res$padj < 0.05 & res$log2FoldChange < -1), ]
write.csv(as.data.frame(up_in_KO), file = "upregulated_in_KO.csv")
```

In [ ]:

```
##############################################################################################################
## For the analysis of enrichment in all DE genes:

################# Gene Ontology (GO) and KEGG pathways analysis using pathfindR

##################################### P. gingivalis analysis #################################################


library(pathfindR)
library(tidyr)
library(dplyr)

descriptions<-read.csv('gsets_KEGG_descriptions_Pg.csv', header = FALSE, row.names = 1)

xy.list <- as.list(as.data.frame(t(descriptions)))

gsets_list<-unlist(xy.list)

custom_descriptions <- gsets_list


###### Generate gene_set

df<-read.csv('STRING_pathwayIDs_Pg_KEGG.csv', header=TRUE)

split_tibble <- function(tibble, column = 'col') {
 tibble %>% split(., .[,column]) %>% lapply(., function(x) x[,setdiff(names(x),column)])
}

dflist<-split_tibble(df, 'group')

custom_genes <- dflist


RA_input<-read.table("RA_input_wt2hcrispr.txt", header = TRUE)

# Now try with this file
RA_processed <- input_processing(input = RA_input,pin_name_path = "pgn_pin_sif_format.sif")

RA_processed_wt2hcrispr<-RA_processed[,2:4]

# Run pathfindR with your custom gene sets and descriptions
results_2h <- run_pathfindR(
  input = RA_processed_wt2hcrispr,  # Use the processed input
  gene_sets = "Custom",
  custom_genes = custom_genes,
  custom_descriptions = custom_descriptions,
  pin_name_path = "pgn_pin_sif_format.sif",
  output_dir = "pathfindR_results_pgn_final",
  p_val_threshold = 0.05,
  enrichment_threshold = 0.05,
  list_active_snw_genes = TRUE
)

# Load and visualize results
enrichment_chart(results_2h, top_terms = 15)
term_gene_graph(results_2h, num_terms = 10)
term_gene_heatmap(results_2h, num_terms = 15)

# Cluster similar terms
clustered_results_2h <- cluster_enriched_terms(results_2h)
enrichment_chart(clustered_results_2h, plot_by_cluster = TRUE)

###################################################################################################

RA_input<-read.table("RA_input_wt6hcrispr.txt", header = TRUE)

# Now try with this file
RA_processed <- input_processing(input = RA_input,pin_name_path = "pgn_pin_sif_format.sif")

RA_processed_wt6hcrispr<-RA_processed[,2:4]

# Run pathfindR with your custom gene sets and descriptions
results_6h <- run_pathfindR(
  input = RA_processed_wt6hcrispr,  # Use the processed input
  gene_sets = "Custom",
  custom_genes = custom_genes,
  custom_descriptions = custom_descriptions,
  pin_name_path = "pgn_pin_sif_format.sif",
  output_dir = "pathfindR_results_pgn_final",
  p_val_threshold = 0.05,
  enrichment_threshold = 0.05,
  list_active_snw_genes = TRUE
)

# Load and visualize results
enrichment_chart(results_6h, top_terms = 15)
term_gene_graph(results_6h, num_terms = 10)
term_gene_heatmap(results_6h, num_terms = 15)

# Cluster similar terms
clustered_results_6h <- cluster_enriched_terms(results_6h)
enrichment_chart(clustered_results_6h, plot_by_cluster = TRUE)

save.image(file="pathfindR_Pg_results.RData")

#########################################################################################

#### Combining results pathfindR Results

combined_df <- combine_pathfindR_results(results_2h,results_6h,plot_common = TRUE)

combined_results_graph(combined_df = combined_df, selected_terms = sample(combined_df$Term_Description), use_description = TRUE, node_size = "p_val", layout = "auto")


##################################### Human analysis #################################################

library(pathfindR)


n_iter <- 10 ## number of iterations

RA_input<-read.table('RA_input_wt6hcrispr.txt', header = TRUE)

RA_processed <- input_processing(input = RA_input, p_val_threshold = 0.05, convert2alias = TRUE)

input_df<-RA_processed[,c(2:4)]

output_df_wt6hcrispr <- run_pathfindR(input_df,gene_sets = "KEGG",min_gset_size = 10, max_gset_size = 300, pin_name_path  = "KEGG", visualize_enriched_terms = TRUE, max_to_plot = 20) ### Top 20 enriched terms


#### Term-gene graph

term_gene_graph(output_df_wt6hcrispr, num_terms = 10, layout = "stress", use_description = FALSE, node_size = "num_genes")

#### Term-gene heatmap

term_gene_heatmap(output_df_wt6hcrispr)


#######################################################################################################

#### Comparison of 2 pathfindR Results #

combined_df <- combine_pathfindR_results(output_df_wt6hcrispr,output_df_wt6h1547)

### Top 10 pathways


combined_results_graph(combined_df, selected_terms = c("hsa04110","hsa04613","hsa04625","hsa04657","hsa05034"))
```

In [ ]:

```
###############################################################################################################################
##### Figure 4c and d and Figure S2.

### In Cytoscape using the ClueGO app. Used for genes present in the different clusters

##### For P. gingivalis analysis on genes identified as hits by SPA.

# Analysis Mode
ClueGO
# Load Marker List(s)
Porphyromonas gingivalis ATCC 33277 [431947]
# Visual Style
Groups
# ClueGO Settings
GO BiologicalProcess-GOA
# Global
# GO Tree interval
3 # Min Level
8 # Max Level
# GO Term/Pathway Selection (#/% Genes)
# Cluster #1
All #Min #Genes
# GO Term/Pathway Network Connectivity (Kappa Score)
High Score: 0.4
# Advance Statistical Options
# GO Term/Pathway Selections
All
Enrichment/Depletion (Two-sided hypergeometric test) ## for enrichment or depletion
Benjamini-Hochberg # pV Correction
# Reference Set options
Selected Ontologies Reference Set
# Grouping Options
# GO Term Grouping (Functional Grouping)
Use GO Term Grouping
# Group coloring
Random
# Leading Group Term based on
Highest Significance
Kappa Score
```

In [ ]:

```
###############################################################################################################################
##### Figure 4a and Figure S4.

#### Pipeline:

# Extract just the regions you want and convert to BED format

    $ awk '$3=="direct_repeat" {print $1"\t"($4-1)"\t"$5}' CRISPR_repeats_regions.gff > crispr_regions.bed

# Use bedtools to mask the regions with N's:

    $ bedtools maskfasta -fi GCF_000010505.1_ASM1050v1_genomic.fna -bed crispr_regions.bed -fo GCF_000010505.1_ASM1050v1_genomic_masked.fna

# Create BWA index for the masked reference

    $ bwa index GCF_000010505.1_ASM1050v1_genomic_masked.fna
    
### We use CRISPR30.1-1 as an example
  
    $ bwa mem -t 34 GCF_000010505.1_ASM1050v1_genomic_masked.fna CRISPR30.1-1.fastq.gz CRISPR30.1-1_R2_001.fastq.gz | samtools view -Sb - > CRISPR30.1-1.bam
    
    $ samtools sort -@ 32 CRISPR30.1-1.bam -o CRISPR30.1-1.sorted.bam
    $ samtools index CRISPR30.1-1.sorted.bam

    $ bedtools genomecov -ibam CRISPR30.1-1.sorted.bam -d > CRISPR30.1-1.coverage.bed

### We used a custom script to call peak with enrichment of hits

##### In peak-caller.py
# --min-height-percentile (default=95): Peaks must be higher than this percentile of coverage values. Higher values (e.g., 99) detect only the tallest peaks.
# --prominence-percentile (default=90): Controls how distinct a peak must be from surrounding regions. Higher values require peaks to stand out more from background.
# --min-peak-distance (default=500): Minimum distance in base pairs between peaks. Larger values prevent calling multiple peaks in close proximity.

    $ python peak-caller.py CRISPR_30.1-1.coverage.bed --normalize --min-height-percentile 99.99 --prominence-percentile 99 --min-peak-distance 1000
    
### We used a custom script to identify what genes or intergenic regions are the hits of the spacers
    
    $ python peak-annotator.py CRISPR_30.1-1.coverage_peaks.bed GCF_000010505.1genomic.fixed.gff > CRISPR_30.1-1.annotated_peaks.bed
```

In [ ]:

```
####################################### peak-caller.py script ####################################

#!/usr/bin/env python3

import sys
import pandas as pd
import numpy as np
from scipy import stats
from scipy.signal import find_peaks, peak_widths
import matplotlib.pyplot as plt
from pathlib import Path
import argparse
from scipy.ndimage import gaussian_filter1d
import statsmodels.stats.multitest as multi

class PeakCaller:
    def __init__(self, window_size=1000, min_peak_distance=500, 
                min_height_percentile=95, prominence_percentile=90,
                smoothing_window=3):
        self.window_size = window_size
        self.min_peak_distance = min_peak_distance
        self.min_height_percentile = min_height_percentile
        self.prominence_percentile = prominence_percentile
        self.smoothing_window = smoothing_window

    def normalize_coverage(self, coverage):
        """Normalize coverage to RPM"""
        total_reads = np.sum(coverage)
        if total_reads > 0:
            return (coverage / total_reads) * 1_000_000
        return coverage

    def smooth_signal(self, coverage):
        """Apply Gaussian smoothing to reduce noise"""
        return gaussian_filter1d(coverage, sigma=self.smoothing_window)

    def call_peaks(self, df, normalize=True):
        """Main peak calling function with optional normalization"""
        peaks_list = []
        
        for chrom, group in df.groupby('chrom'):
            # Convert to numpy arrays for faster processing
            coverage = group['coverage'].to_numpy()
            positions = group['position'].to_numpy()
            
            # Normalize if requested
            if normalize:
                coverage = self.normalize_coverage(coverage)
            
            # Skip if no variation in coverage
            if np.std(coverage) == 0:
                continue
                
            # Smooth signal
            smoothed_coverage = self.smooth_signal(coverage)
            
            # Calculate adaptive thresholds based on data distribution
            min_height = np.percentile(smoothed_coverage, self.min_height_percentile)
            prominence = np.percentile(smoothed_coverage, self.prominence_percentile) * 0.1
            
            try:
                # Find peaks with adaptive thresholds
                peaks, properties = find_peaks(
                    smoothed_coverage,
                    height=min_height,
                    distance=self.min_peak_distance,
                    prominence=prominence
                )
                
                if len(peaks) == 0:
                    continue
                
                # Calculate peak widths
                widths, width_heights, left_ips, right_ips = peak_widths(
                    smoothed_coverage, peaks, rel_height=0.5
                )
                
                # Process each peak
                for i, peak in enumerate(peaks):
                    # Get peak region
                    start_idx = max(0, peak - self.window_size)
                    end_idx = min(len(smoothed_coverage), peak + self.window_size)
                    local_region = smoothed_coverage[start_idx:end_idx]
                    
                    # Calculate local statistics
                    local_mean = np.mean(local_region)
                    local_std = np.std(local_region) + 1e-10
                    zscore = (smoothed_coverage[peak] - local_mean) / local_std
                    
                    # Only keep peaks that are significantly above local background
                    if zscore < 2:  # At least 2 standard deviations above local background
                        continue
                    
                    peaks_list.append({
                        'chrom': chrom,
                        'start': int(positions[int(left_ips[i])]),
                        'end': int(positions[int(right_ips[i])]),
                        'peak_position': positions[peak],
                        'peak_height': smoothed_coverage[peak],
                        'local_enrichment': smoothed_coverage[peak] / (local_mean + 1e-10),
                        'width': widths[i],
                        'zscore': zscore
                    })
            
            except Exception as e:
                print(f"Warning: Error processing chromosome {chrom}: {str(e)}")
                continue
        
        if not peaks_list:
            print("No peaks found that meet the criteria.")
            return pd.DataFrame()
        
        # Create DataFrame and sort by peak height
        peaks_df = pd.DataFrame(peaks_list)
        if not peaks_df.empty:
            peaks_df = peaks_df.sort_values('peak_height', ascending=False)
        
        return peaks_df

    def plot_peaks(self, df, peaks_df, output_file):
        """Plot coverage with called peaks"""
        plt.figure(figsize=(15, 8))
        
        # Plot coverage
        plt.plot(df['position'], df['coverage'], 'b-', alpha=0.5, label='Coverage')
        
        # Highlight peaks
        if not peaks_df.empty:
            plt.scatter(peaks_df['peak_position'], peaks_df['peak_height'], 
                      color='red', s=50, label='Called Peaks')
            
            # Add peak annotations
            for _, peak in peaks_df.iterrows():
                plt.axvspan(peak['start'], peak['end'], 
                          alpha=0.2, color='red')
                # Add position annotation
                plt.annotate(f"pos: {int(peak['peak_position'])}",
                           (peak['peak_position'], peak['peak_height']),
                           xytext=(0, 10), textcoords='offset points',
                           ha='center', va='bottom',
                           fontsize=8, color='red',
                           rotation=45)
        
        # Format x-axis to show positions more clearly
        plt.xlabel('Genomic Position')
        plt.ylabel('Coverage')
        plt.title(f'Coverage Profile with Peak Positions - {df.chrom.iloc[0]}')
        plt.legend()
        
        # Add grid for better readability
        plt.grid(True, alpha=0.3)
        
        # Format x-axis ticks
        plt.ticklabel_format(axis='x', style='plain')  # Prevent scientific notation
        
        # Add vertical lines at peaks for better position visibility
        for _, peak in peaks_df.iterrows():
            plt.axvline(x=peak['peak_position'], color='red', linestyle='--', alpha=0.2)
        
        plt.savefig(output_file, bbox_inches='tight', dpi=300)
        plt.close()

def main():
    parser = argparse.ArgumentParser(description='Advanced Peak Calling from Coverage Data')
    parser.add_argument('input_file', help='Input BED file with coverage data')
    parser.add_argument('--window-size', type=int, default=1000,
                      help='Window size for local background estimation')
    parser.add_argument('--min-peak-distance', type=int, default=500,
                      help='Minimum distance between peaks')
    parser.add_argument('--min-height-percentile', type=float, default=95,
                      help='Percentile of coverage to use as minimum peak height')
    parser.add_argument('--prominence-percentile', type=float, default=90,
                      help='Percentile to use for peak prominence threshold')
    parser.add_argument('--normalize', action='store_true',
                      help='Normalize coverage to RPM before peak calling')
    
    args = parser.parse_args()
    
    # Read input file
    try:
        df = pd.read_csv(args.input_file, sep='\t', 
                        names=['chrom', 'position', 'coverage'])
    except Exception as e:
        print(f"Error reading input file: {e}")
        return
    
    # Initialize peak caller
    peak_caller = PeakCaller(
        window_size=args.window_size,
        min_peak_distance=args.min_peak_distance,
        min_height_percentile=args.min_height_percentile,
        prominence_percentile=args.prominence_percentile
    )
    
    # Call peaks
    print("Calling peaks...")
    peaks_df = peak_caller.call_peaks(df, normalize=args.normalize)
    
    # Generate output files
    input_path = Path(args.input_file)
    base_name = input_path.stem
    
    # Save peaks to BED file
    if not peaks_df.empty:
        output_bed = input_path.parent / f"{base_name}_peaks.bed"
        peaks_df.to_csv(output_bed, sep='\t', index=False)
        print(f"Found {len(peaks_df)} peaks. Saved to {output_bed}")
        
        # Create visualization
        output_plot = input_path.parent / f"{base_name}_peaks.png"
        peak_caller.plot_peaks(df, peaks_df, output_plot)
        print(f"Plot saved to {output_plot}")
    else:
        print("No significant peaks found.")

if __name__ == "__main__":
    main()
```

In [ ]:

```
################################## peak-annotator.py script ##################################

import pandas as pd
import sys
import re

# Check for correct number of arguments
if len(sys.argv) != 3:
    print("Usage: python annotate_peaks.py <peaks_file> <gff_file>")
    sys.exit(1)

# Get input file names from command-line arguments
peaks_file = sys.argv[1]
gff_file = sys.argv[2]

# Load peaks file with the new format
peaks = pd.read_csv(peaks_file, sep="\t")
# If the file doesn't have headers, use these column names
if 'chrom' not in peaks.columns:
    peaks.columns = ["chrom", "start", "end", "peak_position", "peak_height", 
                    "local_enrichment", "width", "zscore"]

# Load GFF file, skipping comment lines
gff = pd.read_csv(gff_file, sep="\t", header=None, comment='#',
                 names=["chrom", "source", "feature", "start", "end", "score", 
                       "strand", "phase", "attributes"])

# Function to extract attributes
def extract_attribute(attributes, key):
    match = re.search(f'{key}=([^;]+)', str(attributes))
    if match:
        return match.group(1)
    return None

# Process gene features
genes = gff[gff["feature"] == "gene"].copy()
genes["locus_tag"] = genes["attributes"].apply(lambda x: extract_attribute(x, "locus_tag"))
genes["gene_name"] = genes["attributes"].apply(lambda x: extract_attribute(x, "Name"))

# Process CDS features
cds = gff[gff["feature"] == "CDS"].copy()
cds["locus_tag"] = cds["attributes"].apply(lambda x: extract_attribute(x, "locus_tag"))
cds["product"] = cds["attributes"].apply(lambda x: extract_attribute(x, "product"))

# Group CDS by locus_tag
cds_products = cds.groupby("locus_tag")["product"].first().reset_index()

# Merge products with genes
genes = genes.merge(cds_products, on="locus_tag", how="left")

# Print some debug information
print(f"Number of genes found: {len(genes)}", file=sys.stderr)
print(f"Number of peaks: {len(peaks)}", file=sys.stderr)
print("Sample of chromosome IDs in peaks:", file=sys.stderr)
print(peaks["chrom"].unique(), file=sys.stderr)
print("Sample of chromosome IDs in genes:", file=sys.stderr)
print(genes["chrom"].unique(), file=sys.stderr)

# Initialize new columns
peaks["type"] = "intergenic"
peaks["locus_tag"] = None
peaks["product"] = None
peaks["flanking_genes"] = None

# Process each peak region
for idx, peak in peaks.iterrows():
    chrom = peak["chrom"]
    peak_pos = peak["peak_position"]  # Use peak position for annotation
    
    # Get genes on the same chromosome
    chrom_genes = genes[genes["chrom"] == chrom].copy()
    
    if not chrom_genes.empty:
        # Find genes that overlap with this position
        overlapping = chrom_genes[
            (chrom_genes["start"] <= peak_pos) & 
            (chrom_genes["end"] >= peak_pos)
        ]
        
        if not overlapping.empty:
            # This peak is within a gene
            gene = overlapping.iloc[0]
            peaks.loc[idx, ["type", "locus_tag", "product"]] = [
                "gene",
                gene["locus_tag"],
                gene["product"]
            ]
        else:
            # Find flanking genes
            upstream = chrom_genes[chrom_genes["end"] < peak_pos]
            downstream = chrom_genes[chrom_genes["start"] > peak_pos]
            
            upstream_gene = upstream.sort_values("end", ascending=False).iloc[0] if not upstream.empty else None
            downstream_gene = downstream.sort_values("start").iloc[0] if not downstream.empty else None
            
            # Format flanking genes information
            upstream_info = (f"{upstream_gene['locus_tag']} ({upstream_gene['product']})" 
                           if upstream_gene is not None and pd.notna(upstream_gene['product'])
                           else "None (None)")
            downstream_info = (f"{downstream_gene['locus_tag']} ({downstream_gene['product']})"
                             if downstream_gene is not None and pd.notna(downstream_gene['product'])
                             else "None (None)")
            
            peaks.loc[idx, "flanking_genes"] = f"{upstream_info} - {downstream_info}"

# Keep all original columns plus the new annotation columns
original_columns = peaks.columns.tolist()
annotation_columns = ["type", "locus_tag", "product", "flanking_genes"]
final_columns = original_columns[:original_columns.index("zscore") + 1] + annotation_columns

# Output results
peaks[final_columns].to_csv(sys.stdout, index=False, sep="\t")
```

In [ ]:

```
####################### Figure 4b - Circos-plot

### Combine all peaks

python combine-peaks.py bed_files/files_with_peaks/ combined_peaks.bed peaks_combined.svg 

################################## combine-peaks.py script ##################################

import pandas as pd
import os
import matplotlib.pyplot as plt
import seaborn as sns
import argparse

## usage: python combine-peaks.py Results_normalized_high_coverage/ combined_peaks.bed peaks_comparison.svg 

def combine_and_plot_peak_files(input_directory, output_file, output_plot):
    """
    Combine multiple peak files and create visualization.
    """
    # Create output directories
    if os.path.dirname(output_file):
        os.makedirs(os.path.dirname(output_file), exist_ok=True)
    if os.path.dirname(output_plot):
        os.makedirs(os.path.dirname(output_plot), exist_ok=True)
    
    # Process peak files
    peak_files = [f for f in os.listdir(input_directory) if f.endswith('_peaks.bed')]
    print(f"Found {len(peak_files)} peak files to process")
    
    all_dfs = []
    for filename in peak_files:
        filepath = os.path.join(input_directory, filename)
        try:
            df = pd.read_csv(filepath, sep='\t')
            df['source_file'] = filename
            all_dfs.append(df)
            print(f"Processed file: {filename}")
        except Exception as e:
            print(f"Error processing {filename}: {e}")
    
    if not all_dfs:
        print("No files were processed successfully")
        return None
    
    # Combine and sort dataframes
    combined_df = pd.concat(all_dfs, ignore_index=True)
    combined_df = combined_df.sort_values(['chrom', 'peak_position'])
    
    # Save combined file
    print(f"Writing combined file to {output_file}")
    combined_df.to_csv(output_file, sep='\t', index=False)
    
    # Create visualization
    plt.figure(figsize=(15, 6))
    color_palette = sns.color_palette("husl", n_colors=len(all_dfs))
    
    for i, df in enumerate(all_dfs):
        plt.scatter(df['peak_position'], df['peak_height'], 
                   label=df['source_file'].iloc[0],
                   color=color_palette[i], 
                   alpha=0.7,
                   s=50)
        
        for _, row in df.iterrows():
            plt.axvspan(row['start'], row['end'], 
                       color=color_palette[i], 
                       alpha=0.2)
    
    plt.title('Peak Positions and Heights Across Samples')
    plt.xlabel('Genomic Position')
    plt.ylabel('Peak Height')
    plt.legend(title='Source Files', bbox_to_anchor=(1.05, 1), loc='upper left')
    plt.grid(True, alpha=0.3)
    plt.tight_layout()
    
    print(f"Saving plot to {output_plot}")
    plt.savefig(output_plot, dpi=300, bbox_inches='tight')
    print("Processing complete!")
    
    return combined_df

def main():
    parser = argparse.ArgumentParser(description='Combine and plot peak files')
    parser.add_argument('input_dir', help='Directory containing peak files')
    parser.add_argument('output_file', help='Path for combined output file')
    parser.add_argument('output_plot', help='Path for output plot')
    
    args = parser.parse_args()
    combine_and_plot_peak_files(args.input_dir, args.output_file, args.output_plot)

if __name__ == "__main__":
    main()
```

In [ ]:

```
############################## Plot Circos-plot for genome map hits in R

> source('circos-plot-fixed-lines.r')

##### circos-plot-fixed-lines.r code 

library(circlize)

peaks <- read.table('combined_peaks.bed', header=TRUE, sep='\t')
genome_length <- 2354887
circos.clear()
circos.initialize("genome", xlim = c(0, genome_length))

circos.trackPlotRegion(
    "genome",
    ylim = c(0, max(peaks$peak_height, na.rm=TRUE)),
    panel.fun = function(x, y) {
        # Using circos.lines instead of segments
        for(i in 1:nrow(peaks)) {
            circos.lines(
                x = c(peaks$peak_position[i], peaks$peak_position[i]),
                y = c(0, peaks$peak_height[i]),
                col = "red",
                lwd = 1  # Increased thickness
            )
        }
        
        circos.points(
            peaks$peak_position,
            peaks$peak_height,
            pch = 16, 
            cex = 1.5,
            col = "red"
        )
        
        for(i in 1:nrow(peaks)) {
            circos.rect(
                peaks$start[i],
                5,
                peaks$end[i],
                peaks$peak_height[i],
                col = adjustcolor("red", alpha=0.2),
                border = NA
            )
        }
        
        min_distance <- 1000
        last_label_position <- -Inf
        sorted_peaks <- peaks[order(peaks$peak_position),]
        
        for(i in 1:nrow(sorted_peaks)) {
            if (sorted_peaks$peak_position[i] - last_label_position >= min_distance) {
                circos.text(
                    sorted_peaks$peak_position[i],
                    sorted_peaks$peak_height[i] + max(peaks$peak_height, na.rm=TRUE)*0.05,
                    labels = formatC(sorted_peaks$peak_position[i], format="d", big.mark=","),
                    facing = "clockwise",
                    niceFacing = TRUE,
                    cex = 0.6,
                    adj = c(0, 0.5)
                )
                last_label_position <- sorted_peaks$peak_position[i]
            }
        }
        
        circos.axis()
    }
)

title("Peak Distribution in Genome")

source_files <- unique(peaks$source_file)
colors <- rainbow(length(source_files))
names(colors) <- source_files

circos.trackPlotRegion(
    "genome",
    ylim = c(0, 1),
    panel.fun = function(x, y) {
        for(i in 1:nrow(peaks)) {
            circos.rect(
                peaks$start[i],
                0,
                peaks$end[i],
                1,
                col = colors[peaks$source_file[i]],
                border = colors[peaks$source_file[i]],
                lwd = 1
            )
        }
    },
    track.height = 0.05,
    bg.border = NA
)

legend("center", 
       legend = sub(".coverage_peaks.bed", "", source_files),
       fill = colors,
       cex = 0.8,
       ncol = 5)

cat("\nSummary Statistics:\n")
print(sample_summary)
```

In [12]:

```
######################################### PCR conditions to confirm ΔCRISPR 30.1 mutant (Fig. S5) ######################################
```

Out[12]:

In [ ]:

```

```
