# Supplementary material for: A CRISPR array orchestrates virulence and host response in Porphyromonas gingivalis
Source: Microbiol Spectr. 2026 Feb 25;14(4):e02834-25. doi: 10.1128/spectrum.02834-25 (PMC13055991; doi:10.1128/spectrum.02834-25)
Supplement: Supplemental legends — Legends for Figures S1 to S5. [file spectrum.02834-25-s0007.docx]

### Supplementary Material

**FIG S1**. **Cytokine and chemokine expression profiles at 2 and 6 hours post-treatment across different experimental groups.** **(A)** Line plots showing the concentration (pg/mL) of various cytokines and chemokines (TNF-α, IL-6, IL-12p70, CXCL1, CXCL2, CXCL8, IL-1α, IL-10, and CXCL9) at 2 hours and 6 hours post-treatment. Data are presented as mean ± standard deviation for each group.Each plot indicates temporal changes and treatment effects on specific immune mediators. **(B)** Heatmaps showing the statistical significance of group comparisons for each cytokine/chemokine at the 2-hour and 8-hour time points. The intensity of pink shading represents the magnitude of statistical significance as measured by the -log10(p-value); darker pink indicates more significant differences, while white/gray indicates less or no statistical significance. Statistical comparisons were conducted between groups, and significance values are color-coded according to the scale bar provided.

**FIG S2**. **Principal component analysis (PCA) of *P. gingivalis* and THP‑1 transcriptomes following infection with wild-type or ΔCRISPR 30.1 strains.** PCA plots visualize global gene expression profiles of biological replicates for each condition, with 95% confidence ellipses encircling replicates. **(A)** PCA of *P. gingivalis* transcriptomes at 2 h and 6 h post-infection, comparing wild-type and ΔCRISPR 30.1 mutant strains. Principal components (PC1 and PC2) capture the most significant variance in bacterial gene expression. **(B)** PCA of THP‑1 cell transcriptomes at 2 h and 6 h post-infection with wild-type or ΔCRISPR 30.1 *P. gingivalis*. Principal components represent the primary axes of variation in host response. Colors denote infection condition (wild‑type vs. mutant) and shapes denote time point (2 h vs. 6 h). Ellipses represent 95% confidence intervals around each group of biological replicates.

**FIG S3**. **PathfindR-derived KEGG pathway enrichment during *P. gingivalis* infection of THP‑1 cells.** Bubble plots display enriched pathways clustered by functional categories. Bubble size represents the number of differentially expressed genes (DEGs) annotated to each pathway. Bubble color indicates the direction of regulation during wild-type infection: red for pathways where DEGs are predominantly up‑regulated and green for pathways where DEGs are predominantly down‑regulated. **(A)** KEGG term enrichment in THP‑1 cells at 2 h and 6 h post-infection with wild-type *P. gingivalis*. **(B)** KEGG term enrichment in *P. gingivalis* at 2 h and 6 h post-infection of THP‑1 cells. In each panel, the x-axis represents fold enrichment, and the y-axis lists KEGG pathway names. Only pathways meeting the enrichment threshold (adjusted *P* < 0.05) are shown.

**FIG S4. Genome mapping of single‑spacer SPA results in *P. gingivalis* ATCC 33277.** Mapped SPA amplicons are plotted along the chromosome; red peaks denote significant, specific binding sites. **(A)** Gel showing an example of results for the SPA experiment. In this case the Tm used was 63^o^C. The 2 lines marked are the samples showed below **(B)** A representative of a positive spacer SPA yields a single, discrete peak, indicating a unique, specific target locus. **(C)** A negative spacer SPA produces widespread, low-amplitude signals across the genome, reflecting non-specific binding to multiple sites.

**FIG S5. Confirmation of the deletion in the mutant ΔCRISPR 30.1. (A)** Schematic diagram describing the strategy to confirm the deletion. **(B)** Results of the 3 PCRs confirming the deletion of the CRISPR 30.1 regions and its substitution by the ermF cassette.
