# Supplementary material for: A CRISPR array orchestrates virulence and host response in Porphyromonas gingivalis
Source: Microbiol Spectr. 2026 Feb 25;14(4):e02834-25. doi: 10.1128/spectrum.02834-25 (PMC13055991; doi:10.1128/spectrum.02834-25)
Supplement: Table S1 — Primers used in the construction and confirmation of the ΔCRISPR-30.1 mutant. [file spectrum.02834-25-s0008.pdf]

Table S1. Primers used in the construction and confirmation of the ΔCRISPR-30.1 mutant.

Primer	5'-3' Sequence	Use
Before CRISPR-30.1-F	ctatgaccatgattacgccaATGAGTGTCTATCGACTCATC	For amplification of before fragment for CRISPR spacer repeat array
Before CRISPR-30.1-R	tagcggaagctatcgggggtacccggcgattggtgtgtattttg	For amplification of before fragment for CRISPR spacer repeat array
ermF with promoter CRISPR-30.1-F	caccaatcgccgGGTACCCCCGATAGCTTC	For amplification of erythromycin cassette with promoter for ΔCRISPR 30.1
ermF with promoter CRISPR-30.1-R	cctatatttgtaacggCTACGAAGGATGAAATTTTTCAGG	For amplification of erythromycin cassette with promoter for ΔCRISPR 30.1
After CRISPR-30.1-F	aatttcatccttcgtagCCGTTACAAATATAGGCTTTTC	For amplification of after fragment for ΔCRISPR 30.1
After CRISPR-30.1-R	ttgtaaaacgacggccagtgTCAGGTTTGTCGGAGAAAG	For amplification of after fragment for ΔCRISPR 30.1
Construct ΔCRISPR 30.1 F primer	ATG AGT GTC TAT CGA CTC ATC CGC	For amplification of entire construct of ΔCRISPR 30.1
Construct ΔCRISPR 30.1 R primer	TCA GGT TTG TCG GAG AAA GCT ATG G	For amplification of entire construct of ΔCRISPR 30.1
External 2.5 kb ΔCRISPR 30.1 F primer	TCC ATC ATG CGT CGC AAG GAA TTG G	For confirmation of clone in *P. gingivalis* ΔCRISPR 30.1
External 2.5 kb ΔCRISPR 30.1 R primer	CCC AAG GCA GGA CGG GAT AAG ATT AT	For confirmation of clone in *P. gingivalis* ΔCRISPR 30.1
M13/pUC Forward	CCC AGT CAC GAC GTT GTA AAA CG	For confirming construct in pUC19 plasmid
M13/pUC Reverse	AGC GGA TAA CAA TTT CAC ACA GG	For confirming construct in pUC19 plasmid
ErmF	CCGATAGCTTCCGCTATTGCTTTTTTGCTCATCGGT	Sequencing of construct
ErmR	ACCGATGAGCAAAAAAGCAATAGCGGAAGCTATCGG	Sequencing of construct
ckErm1-R	CGTAAATGTTCAACCAAAGCTGTG	For confirmation of clone in *P. gingivalis* ΔCRISPR 30.1
qErmF	CCAACTGTCAAATCAGCCCTG	For confirmation of clone in *P. gingivalis* CRISPR 30.1
qErmR	CTTGGAGACAAACAAACAATT	For confirmation of clone in *P. gingivalis* ΔCRISPR 30.1
